# Host FSTL1 defines the impact of stem cell therapy on liver fibrosis by potentiating the early recruitment of inflammatory macrophages

**DOI:** 10.1038/s41392-025-02162-6

**Published:** 2025-03-07

**Authors:** Xiaohong Zheng, Siyuan Tian, Ting Li, Si Zhang, Xia Zhou, Yansheng Liu, Rui Su, Miao Zhang, Bo Li, Chao Qi, Guanya Guo, Shuoyi Ma, Keshuai Sun, Fangfang Yang, Yinan Hu, Chunmei Yang, Lina Cui, Yulong Shang, Changcun Guo, Boquan Jin, Lei Guan, Jingbo Wang, Wen Ning, Ying Han

**Affiliations:** 1 Xijing Hospital of Digestive Diseases, State Key Laboratory of Holistic Integrative Management of Gastrointestinal Cancers, Fourth Military Medical University, Xi’an, China; 2 Department of Biochemistry and Molecular Biology, Fourth Military Medical University, Xi’an, China; 3 State Key Laboratory of Medicinal Chemical Biology, College of Life Sciences, Nankai University, Tianjin, China; 4 Department of Immunology, Fourth Military Medical University, Xi’an, China; 5 Science and Technology Innovation Research Institute, Tangdu Hospital, Fourth Military Medical University, Xi’an, China

**Keywords:** Stem-cell research, Predictive markers

## Abstract

Adult stem cell therapy holds great promise for treating decompensated liver cirrhosis on the basis of animal studies, despite uncertainty about its clinical therapeutic efficacy and unclear underlying mechanisms. Here, we investigated the role of follistatin-like 1 (FSTL1), a profibrotic and proinflammatory matricellular protein, in inflammation-related heterogeneity in stem cell therapy. Our results showed that a high level of circulating FSTL1 is significantly correlated with therapeutic response in patients with cirrhosis. FSTL1 facilitated MSC-mediated early recruitment of Ly6C^+^ inflammatory macrophages within 24 h postinfusion, which was essential for the empowerment of MSCs and subsequent Ly6C^−^CX3CR1^+^ macrophage remodelling at 48 h postinfusion. *Fstl1* deficiency abrogated early macrophage recruitment and effective Ly6C^−^CX3CR1^+^ macrophage accumulation, resulting in the poor antifibrotic effect of MSCs in mice. Whereas, recombinant FSTL1 protein restored the therapeutic efficacy of MSCs in CCl_4_-injured *Fstl1*^*+/−*^ mice. Mechanistically, host FSTL1 enhanced rapid recycling of CCR2 to the membrane via activation of the CD14/TLR4/NF-κB/*ATP6V1G2* axis, leading to early recruitment of Ly6C^+^ monocytes /macrophages. Taken together, our findings revealed that FSTL1 is a critical regulator of the fibrotic immune microenvironment and facilitates subsequent stem cell therapy. These data suggest that FSTL1 could serve as a predictive biomarker of stem cell therapy response in patients with liver cirrhosis.

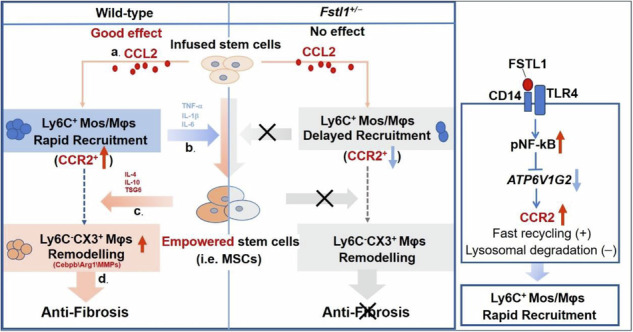

## Introduction

Liver fibrosis caused by multiple aetiologies is a common pathological process of end-stage liver disease and is characterized by inflammatory cell infiltration and excessive collagen deposition. Fibrosis can progress to cirrhosis when not well controlled, which is a major cause of morbidity and mortality worldwide with limited treatment options.^1–3^ Liver transplantation, the only effective clinical strategy for treating end-stage liver diseases to date, is limited by the shortage of available donors.^1,2^ Adult stem cell therapy, especially mesenchymal stem cell (MSC) therapy, holds great promise for treating liver fibrosis according to animal studies.^4^ However, owing to the limited understanding of the treatment mechanisms involved, clinical trials of these therapies have not shown definite efficacy.^5–7^

The therapeutic potential of MSCs is predominantly dependent on their strong paracrine capacity and their immunosuppressive effect on immune cells.^6^ A series of studies revealed that MSC-mediated macrophage phenotype modulation contributed to fibrosis resolution.^6^ Macrophage depletion ameliorates the therapeutic effect on liver fibrosis, indicating the indispensable role of macrophages in MSC therapy.^8,9^ However, there is no consensus on the MSC-mediated effective subset of macrophages. Recent studies have shown that the C-X3-C motif chemokine receptor 1 positive (CX3CR1^+^) subset is involved in stem cell therapy in both clinical and preclinical research,^10–12^ in addition to the restorative lymphocyte antigen 6 family member C negative (Ly6C^−^) subset.^6,13^

The immunosuppressive activity of MSCs is determined by a myriad of inflammatory mediators under certain pathological conditions.^14–17^ Immunosuppressants alter the established inflammatory tissue microenvironment of infused MSCs, leading to highly precarious outcomes of clinical therapies.^18,19^ How the inflammatory microenvironment during liver fibrogenesis affects the efficacy of stem cell therapy has not been fully extensively investigated. In the fibrotic microenvironment, macrophages are also key drivers of hepatic inflammation and are the main sources of inflammatory mediators during fibrogenesis.^20^ In mice, Ly6C^+^ monocytes quickly respond to inflammatory signals and migrate to the inflamed liver in a C-C motif chemokine ligand 2 (CCL2)/ C-C motif chemokine receptor 2 (CCR2)-dependent manner, where they differentiate into highly proinflammatory and fibrogenic macrophages (Ly6C^+^).^21^ In patients, CD14^+^ monocyte activity, which highly dynamically and progressively changes in parallel to the cirrhosis stage, can reflect cirrhosis-associated inflammation.^22^ Previous studies revealed that MSCs recruit C-X-C motif chemokine receptor 3 positive (CXCR3^+^) T cells in a chemokine-dependent manner to increase their immunosuppressive capacity.^19^ Recently, Vagnozzi, R.J. et al. reported that intracardiac injection of stem cells induced the temporal accumulation of CCR2^+^ and CX3CR1^+^ macrophages, which highlights the benefit of the treatment effect.^10^ However, whether inflammatory macrophages affect the treatment efficacy of liver fibrosis has still not been reported.

FSTL1, an extracellular glycoprotein highly expressed during liver fibrosis, regulates the inflammatory macrophage phenotype and participates in fibrogenesis.^23–27^ It was initially identified as a TGF-β-inducible secreted glycoprotein belonging to the secreted protein acidic and rich in cysteine (SPARC) family,^28,29^ and functions as a modulator of cell‒matrix interactions by integrating signalling networks to participate in pathological conditions.^23,24,30,31^ During hepatic fibrogenesis, macrophage-derived FSTL1 promotes liver fibrosis by reprogramming M1 macrophage function.^24^ In addition, hepatic stellate cell (HSC)-derived FSTL1 facilitates myofibroblast activation via the transforming growth factor β (TGF-β)/Smad signalling pathway.^23,32,33^ Research has shown that plasma FSTL1 potentially serves as a noninvasive diagnostic biomarker for patients with advanced liver fibrosis.^34^ Moreover, the role of FSTL1 in the treatment of MSCs has also been reported in a myocardial ischaemia model.^35^ We previously reported that MSC-derived FSTL1 affects the immunosuppressive capacity and treatment efficacy of MSCs in liver fibrosis.^36^ Recently, we found the liver cirrhosis patients with high levels of FSTL1 respond to stem cell therapy, but the role of FSTL1 in the fibrotic microenvironment during stem cell therapy has not been determined. To this end, we aimed to study whether host FSTL1 affects the therapeutic efficacy of stem cells in liver fibrosis by modulating the fibrotic microenvironment in which stem cells are exposed. Using *Fstl1-*deficient mice, we found that *Fstl1* deficiency abrogated the treatment effect on stem cells. By analysing the single-cell transcriptome profile of mouse fibrotic livers after MSC transplantation, we identified an increased number of cells of the effective macrophage subset (Ly6C^−^CX3CR1^+^) in wild-type mice and an insufficient number of cells of this subset in *Fstl1-*deficient mice. Furthermore, we revealed that FSTL1 promoted MSC-mediated early recruitment of macrophages, which was indispensable for the immunosuppressive capacity of MSCs under fibrotic conditions. Mechanistically, FSTL1 facilitated the fast recycling of CCR2 via inhibition of *ATP6V1G2* expression and lysosomal degradation of CCR2 through the CD14/TLR4/NF-κB axis. Our data suggest that FSTL1 promotes early macrophage recruitment to facilitate subsequent effective macrophage accumulation during stem cell therapy, and could potentially serve as a predictive biomarker of the response to stem cell therapy in patients with liver cirrhosis.

## Results

### Serum FSTL1 is associated with the clinical response to stem cell therapy in patients with liver cirrhosis

**Fig. 1:**
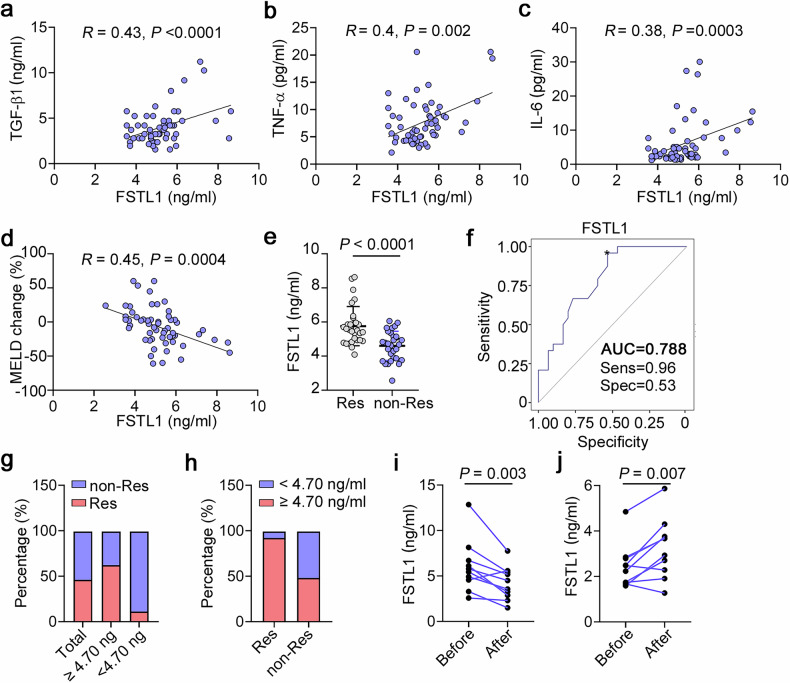
Correlation analysis of serum FSTL1 with inflammatory cytokines and the response to autologous stem cell therapy in patients with liver cirrhosis. Correlation analysis of FSTL1 and fibrotic cytokines, including (**a**) TGF-β, (**b**) TNF-α, and (**c**) IL-6 (*n* = 58). **d** MELD change ratio according to the FSTL1 concentration (*n* = 58). **e** Serum FSTL1 in the MSC-responsive (*n* = 27) and MSC-nonresponsive groups (*n* = 31) determined by a magnetic Luminex assay (*n* = 58). **f** ROC curves with data comparing responsive (*n* = 27) and nonresponsive patients (*n* = 31). **g** Response ratios of the FSTL1^high^ (≥4.7 ng/mL) and FSTL1^low^ (<4.7 ng/mL) groups. **h** Ratios of FSTL1^high^ and FSTL1^low^ patients among responders (**i**) and nonresponders (**j**). Serum FSTL1 levels were evaluated in responders (*n* = 9) and nonresponders (*n* = 9) at 6 months after cell transplantation. Statistical significance was determined by the Spearman rank correlation test (**a**–**d**), Mann–Whitney U test (**e**), and Wilcoxon’s matched-pairs test (**i**, **j**). Error bars indicate the mean ± SEM. Res, response; non-Res, nonresponse

Consistent with previous findings,^23^ we observed that the FSTL1 levels were elevated in the serum of cirrhosis patients (Supplementary Fig. 1a) and in carbon tetrachloride (CCl_4)_-induced model mice (Supplementary Fig. 1b) and were positively correlated with the serum TGF-β1, TNF-α and IL-6 levels in cirrhosis patients (Fig. 1a–c). Before investigating the role of FSTL1 in stem cell therapy, we first verified its expression in two published single-cell gene profiling datasets (Gene Expression Omnibus, GEO), namely, liver nonparenchymal cell types from cirrhotic patients (GSE136103) and a CCl_4_-induced mouse model (GSE171904). We found that *FSTL1*-positive cells were distributed mainly in the mesenchyme and endothelial clusters (Supplementary Fig. 2a, b), but were significantly more common in the mesenchyme in cirrhotic livers (Supplementary Fig. 2c). Further clustering of human liver mesenchymal cells revealed four populations (Supplementary Fig. 2d, e). *FSTL1* was highly expressed in the Mes cluster (4), which was distinguished by PDGFRΑ and COL1A1 expression (Supplementary Fig. 2f–h). We subsequently explored *Fstl1* expression in a CCl_4_-induced mouse model (GSE171904). The results revealed that *Fstl1* was highly expressed in HSCs, portal fibroblasts (PFs) and cholangiocytes in CCl_4_-treated livers (Supplementary Fig. 2i, k).

To verify the correlation between therapeutic efficacy and the FSTL1 level, we recruited 58 patients with liver cirrhosis receiving stem cell transplantation (Supplementary Table 1). Correlation analyses revealed that the baseline serum level of FSTL1 was positively correlated with improvements in the model for end-stage liver disease (MELD) score (R = 0.45) (Fig. 1d). In addition, we detected higher FSTL1 levels in the responder group (5.7 ± 0.3 ng/ml) than in the nonresponder group (4.6 ± 0.6 ng/ml) (Supplementary Fig. 3, Fig. 1e). To evaluate the ability of FSTL1 to predict treatment efficacy, we performed standard receiver operating characteristic (ROC) curve analyses. The results revealed that FSTL1 had an area under the curve (AUC) of 0.788, with an optimal cut-off of 4.7 ng/ml (Fig. 1f). Among FSTL1^hi^ patients (≥4.7 ng/ml), the response/nonresponse ratios were 62.5/37.5 (25/15), while the ratios among the whole cohort and serum FSTL1^lo^ patients (<4.7 ng/ml) were 46.5/53.5 (27/31) and 11.1/88.9 (2/16), respectively (Fig. 1g) In turn, the FSTL1^hi^/FSTL1^lo^ ratios in responders and nonresponders were 92.6/7.4 (25/2) and 48.4/51.6 (15/16), respectively (Fig. 1h). These data indicate that a high baseline serum level of FSTL1 may indicate a better treatment response. Considering its pathological effect during fibrosis, the serum levels of FSTL1 were evaluated 6 months after stem cell transplantation. The results revealed that FSTL1 expression was significantly decreased in responders (Fig. 1i) but not in nonresponders (Fig. 1j). Taken together, our findings suggest that the serum level of FSTL1 is associated with the clinical response of liver cirrhosis patients to stem cells.

### Recipient FSTL1 facilitates stem cell therapy in liver fibrosis

**Fig. 2:**
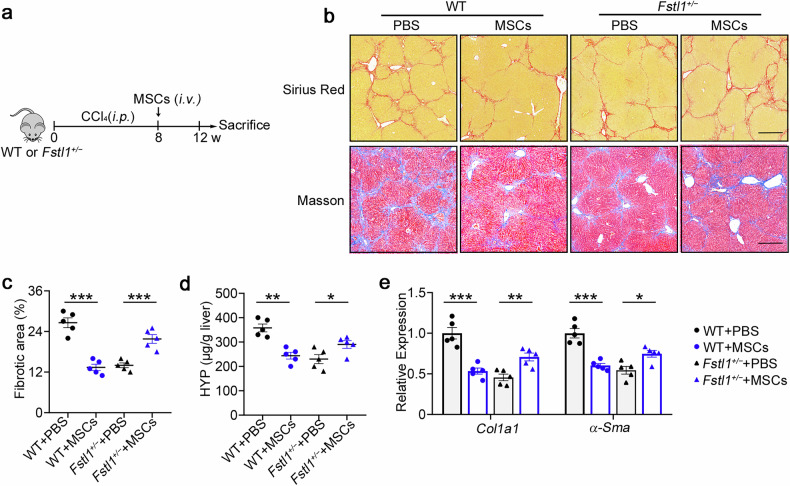
Antifibrotic effects of stem cells are abrogated in *Fstl1*^*+/*−^ mice. **a** Schematic illustration of hepatic fibrosis model establishment and the MSC-based treatment strategy. **b**–**e** The fibrosis degree was evaluated in *Fstl1*^*+/*−^ (*n* = 5) and WT littermates (*n* = 5) at 4 weeks after cell infusion. **b** Liver sections were stained with Sirius red or Masson’s trichrome. Representative images of the staining are shown. Bars, 200 µm. **c** Liver fibrosis score analysis of Sirius red-stained liver sections. The fibrotic area is presented as a percentage. **d** The concentrations of hydroxyproline (HYP) in liver homogenates were determined. *p < 0.05, **p < 0.01, and ***p < 0.001. Statistical significance was determined by one-way ANOVA with Tukey multiple comparison test (**c**, **d**) or two-way ANOVA with Tukey multiple comparison test (**e**). Data are presented as the mean ± SEM and were pooled from at least three independent experiments

To further determine the role of FSTL1 in stem cell-based therapy, we compared the treatment effects in wild-type and *Fstl1*^*+/−*^ mice (Fig. 2a). Surprisingly, these *Fstl1*^*+/−*^ mice no longer benefited from MSC therapy (Fig. 2b, e). Sirius red and Masson staining of liver sections (Fig. 2b, c) and hydroxyproline (HYP) levels in the liver (Fig. 2d) revealed no obvious amelioration of fibrosis in *Fstl1*^+/−^ mice at 4 weeks after MSC transplantation. Consistent with these results, collagen accumulation and myofibroblast activation (as indicated by *Col1* and *α-Sma* expression, respectively) were not obviously improved in the liver tissues of *Fstl1*^*+/−*^ mice (Fig. 2e). These studies suggest that *Fstl1*^*+/−*^ mice cannot benefit from MSC therapy.

To further verify the role of FSTL1 in stem cell therapy, we isolated bone marrow-derived mononuclear cells (MNCs), another cell type extensively used in human clinical trials, and evaluated their therapeutic effect in a CCl_4_-induced model (Supplementary Fig. 4a). Consistent with the results of MSC-based therapy, we also found that MNCs significantly ameliorated liver fibrosis in wild-type mice but showed no functional benefit on liver fibrosis in *Fstl1*^*+/−*^ mice (Supplementary Fig. 4b–e). These data indicate that FSTL1 can affect the treatment effect of stem cells on liver fibrosis.

### FSTL1 facilitates stem cell-mediated Ly6C^*−*^CX3CR1^+^ subset remodelling

**Fig. 3:**
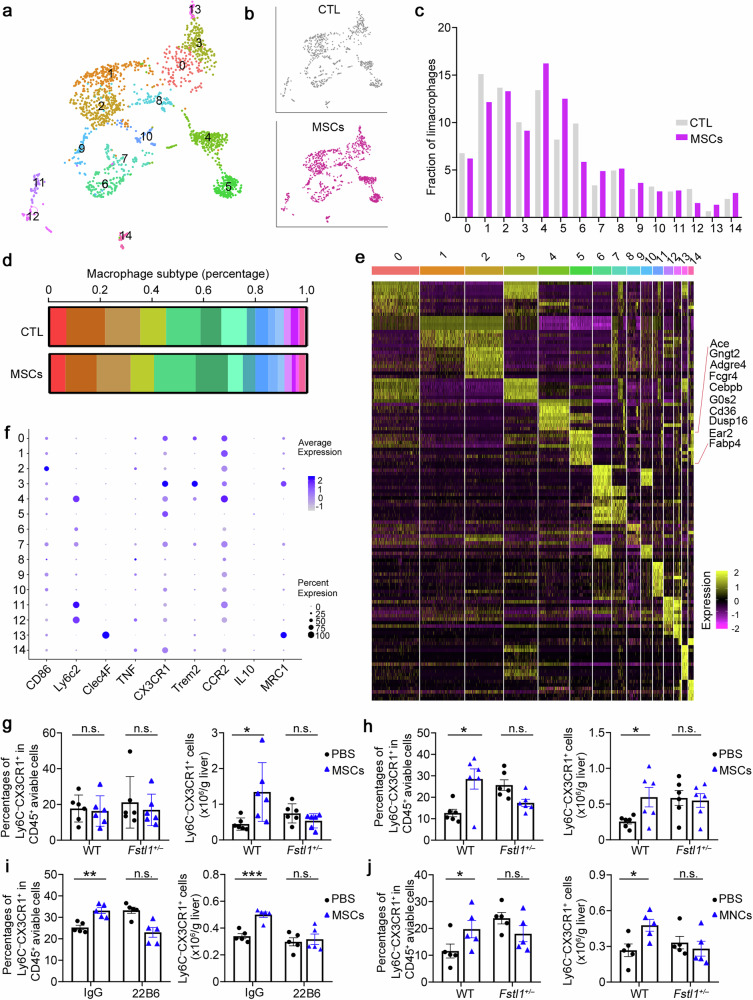
FSTL1 facilitates stem cell-mediated Ly6C^*−*^CX3CR1^+^ subset remodelling. **a** UMAP analysis of 4300 macrophages from 3 PBS- and 3 MSC-treated mice identified 15 distinct cell clusters. **b** Cluster distribution of cells in PBS- (gray) and MSC- (purple) treated mice. **c** Comparison of the populations of different cell lineages between PBS-treated and MSC-treated livers. **d** The relative percentages of different cell lineages in each group, coloured according to cell lineage. PBS: *n* = 3 liver samples from PBS-treated mice; MSC: *n* = 3 liver samples from MSC-treated mice. **e** Heatmap displaying the relative expression levels of marker genes in 15 cell lineages (top, colour-coded by cell lineage), with exemplar genes labelled in Cluster 5. The columns denote cells; the rows denote genes. **f** Marker expression in different clusters. **g**–**j** The percentages of the Ly6C^−^CX3CR1^+^ subset among the total viable hepatic CD45^+^ cells and the cell count of the Ly6C^−^CX3CR1^+^ subset were normalized to the liver weight, as determined by flow cytometry. **g**, **h** The Ly6C^−^CX3CR1^+^ subset was evaluated in *Fstl1*^*+/*−^ (*n* = 6) and WT littermates (*n* = 6) at 24 h (**g**) and 48 h (**h**) after MSC infusion. **i** The Ly6C^−^CX3CR1^+^ subset was evaluated in C57BL/6 mice treated with (*n* = 5) or without the FSTL1 neutralizing antibody 22B6 (*n* = 5) at 48 h after MSC infusion. **j** The Ly6C^−^CX3CR1^+^ subset was evaluated in *Fstl1*^*+/*−^ (*n* = 5) and WT littermates (*n* = 5) at 48 h after MNC infusion. *p < 0.05, **p < 0.01, ***p < 0.001, and n.s., not significant. Statistical significance was determined by a two-tailed unpaired *t*-test (**g**–**j**). Data are presented as the mean ± SEM and were pooled from at least three independent experiments

Before further evaluation of how FSTL1 affects the antifibrotic effect of MSCs, we performed 3′-scRNA-seq analyses of liver cells from the PBS- and MSC-treated groups to determine the subset of effective macrophages. Considering the immunomodulatory property of MSCs in fibrosis and their short survival in the host, we sorted viable hepatic CD45^+^ cells at 48 h postinfusion in both groups (time points also reported elsewhere)^37^ (Supplementary Fig. 5a). The data indicated that 15325 cells were obtained from the PBS (7785 cells) and MSC groups (7540 cells) after quality control (Supplementary Fig. 5a–c). After dimension reduction and clustering analysis, uniform manifold approximation and projection (UMAP) revealed 11 cell lineages (Supplementary Fig. 5b), each containing cells from PBS- and MSC-treated mice. These cell lineages expressed specific marker genes (Supplementary Fig. 5c) and significant differences in different cell subtypes between the two groups are shown (Supplementary Fig. 5d). As macrophages play an indispensable role in the resolution of fibrosis, we further analysed subsets of macrophages (1896 cells). Clustering analysis of the 3′-scRNA-seq data revealed that macrophages formed 15 clusters 0 to 14 across all the subjects (Fig. 3a, b). Importantly, a unique macrophage cluster, cluster 5, was identified, which was obviously increased in the MSC-treated group (Fig. 3c, d). Notably, high levels of *Cebpβ* and angiotensin-converting enzyme (*Ace*) expression were detected in cluster 5 (Fig. 3e). As shown in Fig. 3f, this subset highly expressed *Cx3cr1 and* expressed low levels of *Ly6c* and *Ccr2*. FCM analysis revealed that the Ly6C^*−*^CX3CR1^+^ subset was positive for Cebpβ and negative for CCR2 (Supplementary Fig. 5e), and highly expressed matrix metalloproteinases (*Mmps*) and *Arg1* (Supplementary Fig. 5f, g), suggesting that this subset has antifibrotic and immunosuppressive effects on MSC-mediated fibrotic resolution. The Ly6C^*−*^CX3CR1^+^ subset in bone marrow-derived macrophages (BMDMs) was increased after cocultured with MSCs in vitro (Supplementary Fig. 5h), which was consistent with the upregulation of related genes (*Cebpβ*, *Arg1*, and *Mmps*; Supplementary Fig. 5i). On the basis of the results of the scRNA-seq analysis above, we further confirmed this subset by FCM at 12–48 h post-transplantation in WT and *Fstl1*^+/*−*^ mice (Fig. 3g, h). We observed an obvious increase in the Ly6C^*−*^CX3CR1^+^ subset in WT mice at 48 h (Fig. 3h) beginning at 24 h postinfusion (Fig. 3g). However, this increase was not observed in *Fstl1*^+/*−*^ mice, which probably caused no improvement after MSC therapy (Fig. 3g, h; Supplementary Fig. 5j, k). The same results were observed in the FSTL1 neutralizing antibody (22B6)-treated mice 48 h after MSC infusion, which further confirmed the role of FSTL1 in MSC-mediated Ly6C^*−*^CX3CR1^+^macrophage remodelling (Fig. 3i). As shown in Fig. 3j, MNCs also exerted their immunosuppressive effect at 48 h postinfusion in wild-type mice, as indicated by the accumulation of the Ly6C^*−*^CX3CR1^+^ subset, which was not observed in *Fstl1*^+/*−*^ mice. Consistently, FSTL1 deficiency similarly abolished the early immunosuppressive effect of stem cell transplantation in fibrotic mice.

### Ly6C^+^ macrophage recruitment after cell infusion likely facilitates stem cell-mediated Ly6C^*−*^CX3CR1^+^ macrophage remodelling

**Fig. 4:**
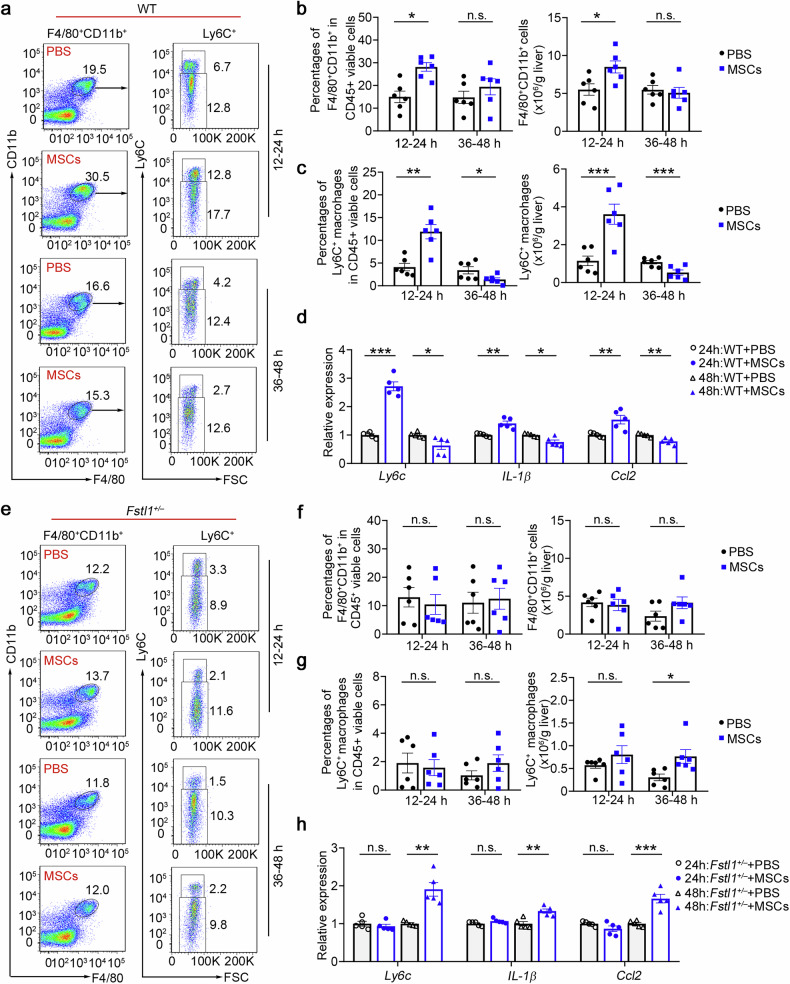
Early rapid recruitment of Ly6C^+^ macrophages after cell infusion is observed in the wild type. **a** Macrophage infiltration was evaluated in the wild type (*n* = 6) at 24 h and 48 h after MSC infusion via flow cytometry. **b** The percentages of F4/80^+^CD11b^+^ cells among the total viable CD45^+^ cells and the cell count normalized to the liver weight were determined. **c** The percentages of Ly6C^+^ macrophages in the F4/80 CD11b^+^ population were compared, and the cell count was normalized to the liver weight. **d** Macrophages were isolated from the livers of WT mice treated with (*n* = 6) or without (*n* = 6) MSCs at 48 h postinfusion. mRNA levels were determined by qPCR. **e** Macrophage infiltration was evaluated in *Fstl1*^*+/*−^ cells (*n* = 6) at 24 h and 48 h after MSC infusion by flow cytometry. **f** The percentages of F4/80^+^CD11b^+^ cells among the total viable CD45^+^ cells and the cell count normalized to the liver weight were determined. **g** The percentages of Ly6C^+^ macrophages in the F4/80^+^CD11b^+^ population were compared, and the cell count was normalized to the liver weight. **h** Macrophages were isolated from the livers of *Fstl1*^*+/*−^ mice treated with (*n* = 5) or without (*n* = 5) MSCs at 48 h postinfusion. mRNA levels were determined by qPCR. *p < 0.05, **p < 0.01, ***p < 0.001, and n.s., not significant. Statistical significance was determined by a two-tailed unpaired *t*-test (**b**, **c**, **f**, **g**) and two-way ANOVA with Tukey multiple comparison test (**d**, **h**). Data are presented as the mean ± SEM and were pooled from at least three independent experiments

As multiple in vitro studies have indicated that inflammatory stimuli are essential for MSC therapy,^14–17^ we next explored how the fibrotic microenvironment affects their treatment effect. As shown in Fig. 4a, c, we surprisingly found that infiltrating macrophages and Ly6C^+^ macrophages were significantly recruited at 12–24 h after MSC transplantation, which was consistent with the accumulation of circulating monocytes at the same time point (Supplementary Fig. 6a). CCL2 was detected in the supernatants of MSCs/MNCs and C57BL/6 mouse serum after MSC transplantation (Supplementary Fig. 6b), indicating that the recruitment of hepatic monocyte-derived macrophages is mediated by transplanted stem cells. There was an obvious reduction in the number of inflammatory macrophages (Ly6C^+^) at 36–48 h after cell infusion in the wild type, whereas the number of total liver infiltrating macrophages (F4/80^+^CD11b^+^) did not significantly change (Fig. 4c). Gene expression in macrophages further validated these results (Fig. 4d). Although we found that infiltrating macrophages and Ly6C^+^ macrophages were significantly recruited at 12–24 h postinfusion in wild-type mice, we did not observe significantly rapid recruitment in *Fstl1*^*+/−*^ mice (Fig. 4e–h). In addition, neither a reduction in the number of inflammatory Ly6C^+^ nor total liver infiltrating macrophages was observed in *Fstl1-*deficient mice 48 h postinfusion. Hence, we propose that FSTL1 likely facilitates stem cell-mediated Ly6C^−^CX3CR1^+^ macrophage remodelling by promoting Ly6C^+^ macrophage recruitment after cell infusion.

### Inflammatory macrophages reprogram metabolism and improve the immunosuppressive capacity of MSCs

**Fig. 5:**
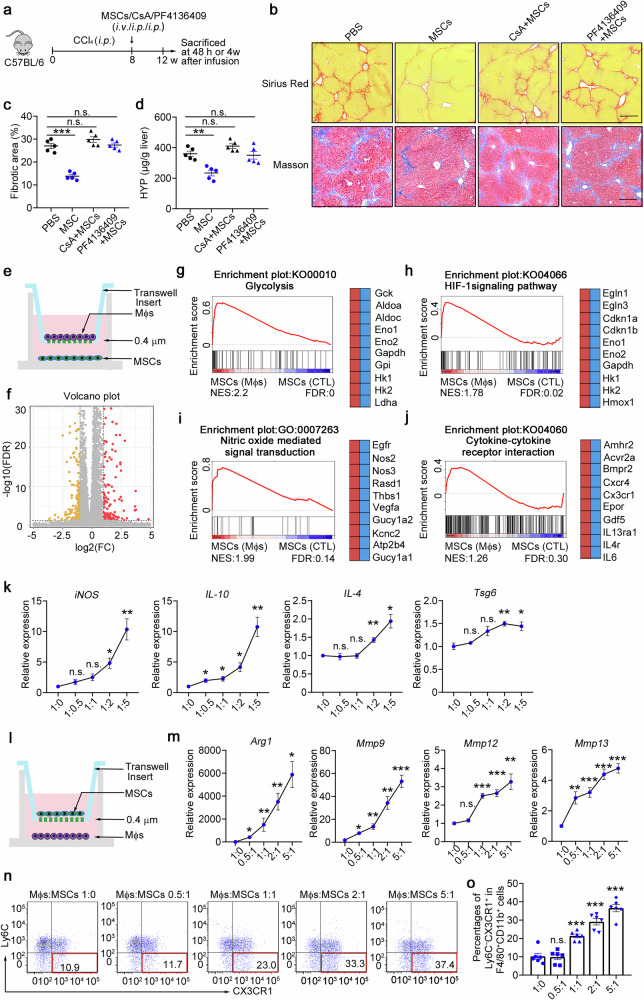
Inflammatory macrophages reprogram metabolism and improve the immunosuppressive capacity of MSCs. **a** Schematic illustration of the hepatic fibrosis model establishment and the MSC-based treatment strategy. **b**–**d** The degree of fibrosis was evaluated in the PBS-, MSC-, CsA+MSC-, and PF4136409 + MSC-treated groups at 4 weeks postinfusion (*n* = 5). **b** Liver sections were stained with Sirius red or Masson’s trichrome. Representative images of the staining are shown. Bars, 200 µm. **c** Liver fibrosis score analysis of Sirius red-stained liver sections. The fibrotic area is presented as a percentage. **d** The concentrations of hydroxyproline (HYP) in liver homogenates were determined. **e** Schematic of the in vitro coculture system for MSCs and BMDMs. **f** Volcano plot showing genes whose expression changed in MSCs cocultured with BMDMs compared with control MSCs, as determined by RNA-seq (*n* = 4). **g**–**j** GSEA plots (left) and heatmaps (right) of the RNA-seq data of MSCs cocultured with BMDMs and control MSCs. Representative genes from each category are shown. FDR, false discovery rate; NES, normalized enrichment score. *n* = 4 mice per group. Statistical significance was determined by linear modelling and Bayesian statistics after correcting for multiple testing with the Benjamini–Hochberg procedure (**f**) or the Wald test with Benjamini–Hochberg’s multiple-comparison correction (**g**–**j**). **k** Gene expression was determined by qPCR in MSCs cocultured with increasing amounts of BMDMs (*n* = 4). **l** Schematic of the in vitro coculture system for MSCs and BMDMs. **m** Gene expression was determined by qPCR in altered amounts of BMDMs cocultured with MSCs (*n* = 4). **n**, **o** The percentages of the Ly6C^−^CX3CR1^+^ subset were determined via flow cytometry in altered amounts of BMDMs after coculture with MSCs (*n* = 6). *p < 0.05, **p < 0.01, ***p < 0.001, and n.s., not significant. Statistical significance was determined by one-way ANOVA with Tukey multiple comparison test (**k**, **m**, **o**). Data are presented as the mean ± SEM and were pooled from at least three independent experiments

Given that strong inflammatory conditions are essential for MSC empowerment,^14–17^ we hypothesized that early recruitment of Ly6C^+^ macrophages is an essential inflammatory condition that promotes their immunosuppressive capacity under chronic inflammation during fibrosis. To further demonstrate the effect of inflammatory macrophages on MSC efficacy in vivo, we used a CCl_4_-induced model treated with cyclosporine A (CsA, a broad-spectrum immunosuppressant) or a CCR2 inhibitor (PF4136409) to block Ly6C^+^ inflammatory macrophage recruitment before MSC transplantation (Fig. 5a). As shown in Fig. 5b–d, MSCs-combined with CsA pretreatment or PF4136409 pretreatment had significantly less beneficial effects. Sirius red and Masson staining of liver sections (Fig. 5b, c) and hydroxyproline (HYP) levels (Fig. 5d) revealed that CsA and PF4136409 pretreatment abolished the antifibrotic effect of MSCs without alleviating extracellular matrix (ECM) deposition. Neither a reduction in the number of inflammatory Ly6C^+^ cells nor an increase in the number of cells of the Ly6C^*−*^CX3CR1^+^ subset was observed in these two pretreatment groups (Supplementary Fig. 7a–d). These data indicate that the proinflammatory microenvironment before MSC infusion and early inflammatory macrophage recruitment determine the immunosuppressive function and final treatment effect of MSCs.

Hence, we hypothesized that stem cell-derived CCL2 signalling to recruit Ly6C^+^ macrophages provides a critical microenvironment for determining the immunosuppressive capacity of MSCs and has a profound effect on the treatment of liver fibrosis. We then investigated how inflammatory macrophages licence MSCs. To compare the transcriptome profiles of MSCs and macrophage-educated MSCs, we extracted RNA and performed quantitative RNA sequencing (RNA-seq) of each sample. We detected 398 differentially expressed genes in total; among these genes, 145 genes were significantly downregulated and 116 genes were upregulated in the macrophage-educated MSCs compared with the control MSCs (Fig. 5e, f, Supplementary Fig. 8a). Gene set enrichment analysis (GSEA) revealed that the upregulated genes in macrophage-educated MSCs were highly enriched in glycolysis metabolic pathways (Fig. 5g, such as *Hk2* and *Ldha*), the HIF-1α signalling pathway (Fig. 5h) and the cellular response to decreased oxygen levels (Supplementary Fig. 8b). Moreover, the upregulated genes included many immunosuppressive genes (such as *Nos2/iNOS*, *Nos3* and *Thbs1*) and were highly enriched in nitric oxide-mediated signal transduction genes (Fig. 5i, Supplementary Fig. 8c, d). In addition, the macrophage-educated MSCs highly expressed genes involved in cytokine‒cytokine receptor interactions (Fig. 5j, such as *Cxcr4* and *IL-6*) and growth factor activity (Supplementary Fig. 8e). The expression of genes related to the 'complement and coagulation cascade' (Supplementary Fig. 8f) and 'regulation of the mesenchymal cell apoptotic process' (Supplementary Fig. 8g) also increased in macrophage-educated MSCs, suggesting that inflammatory macrophages could also promote the MSCs apoptotic process and participate in immunomodulation. To better understand the effects of macrophages on the effect of MSC treatment, MSCs were cocultured with BMDMs at various ratios. Reverse transcription PCR (RT–PCR) analyses revealed that a relatively high ratio of inflammatory macrophages/MSCs potentially induced the expression of immunosuppressive genes in MSCs (*iNOS*, *IL-10*, *IL-4* and *Tsg6*; Fig. 5k). On the other hand, high proportions of macrophages effectively increased the expression of *Arg-1*, *Mmp9* and *Mmp13* (Fig. 5l, m) and significantly increased the number of Ly6C^*−*^CX3CR1^+^ cells (Fig. 5n, o) after cocultured with MSCs. Taken together, these data indicate that inflammatory macrophages reprogram metabolism and improve the immunosuppressive capacity of MSCs.

### FSTL1 potentiates Ly6C^+^ macrophage hepatic recruitment by upregulating CCR2 expression

**Fig. 6:**
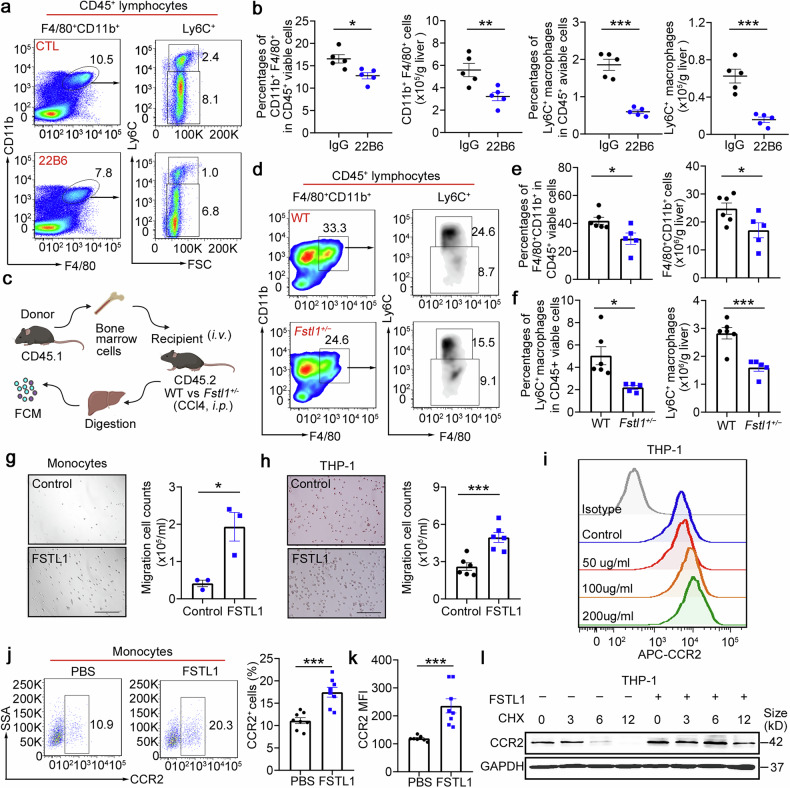
FSTL1 potentiates Ly6C+ macrophage hepatic recruitment. **a**, **b** Macrophage infiltration was evaluated by flow cytometry in C57BL/6 mice (*n* = 5) after 8 weeks of CCl4 induction with or without 22B6 treatment (**a**). **b** The percentages of F4/80^+^CD11b^+^ cells among the total viable CD45^+^ cells and the cell counts normalized to the liver weight were determined. The percentages of Ly6C+ macrophages among the total viable CD45^+^ cells and the cell counts were normalized to the liver weight. **c** Schematic illustration of the adoptive transfer of CD45.1 bone marrow cells into CD45.2 *Fstl1*^*+/*−^ mice. **d**–**f** CD45.1^+^ infiltrated macrophages (**d**, **e**) and CD45.1^+^Ly6C^+^ macrophages (**f**) were evaluated 48 h post injection (*n* = 5). **g** Transwell assays were used to evaluate monocyte-mediated CCL2-induced chemotaxis in cells treated with or without FSTL1 (*n* = 3). **h** Transwell assays were used to evaluate CCL2-induced chemotaxis in THP-1 cells with or without FSTL1 treatment (*n* = 5). Bars, 200 µm (**g**, **h**). **i** CCR2 expression in THP-1 cells was evaluated via FCM with the indicated concentrations of FSTL1. **j**, **k** CCR2 expression in monocytes was evaluated via FCM after FSTL1 treatment (*n* = 8). **l** CHX (10 μg/ml) was used to inhibit protein synthesis. CCR2 expression was evaluated at the indicated time points in the presence or absence of FSTL1. *p < 0.05, **p < 0.01, ***p < 0.001. Statistical significance was determined by a two-tailed unpaired *t*-test (**b**, **e**, **f**–**h**, **j**, **k**). Data are presented as the mean ± SEM and were pooled from at least three independent experiments

We wondered whether FSTL1 facilitates the immunosuppressive function of stem cells by mediating early inflammatory macrophage recruitment. We found that TNF-α, IL-1β and IL-6 were decreased in *Fstl1*^*+/−*^ mice (Supplementary Fig. 9a–d). A reduction in the number of infiltrating monocytes/macrophages and in the proportion of profibrotic cells (Ly6C^*+*^*)* were observed in *Fstl1*-deficient mice (Supplementary Fig. 9e–j). We then blocked FSTL1 function in the wild-type strain with a neutralizing antibody (22B6) and observed the same reduction in total macrophages and the profibrotic proportion of macrophages 48 h after 22B6 treatment (Fig. 6a, b).

To further clarify the role of FSTL1 in macrophage infiltration, we adoptively transferred CD45.1 bone marrow cells into *Fstl1*^*+/−*^ mice via intraperitoneal injection and evaluated the numbers of infiltrating CD45.1^+^ macrophages and infiltrating CD45.1^+^Ly6C^+^ macrophages at 48 h postinjection (Fig. 6c). A reduction in the number of infiltrating CD45.1^+^ macrophages and the Ly6C^+^ subset was observed in *Fstl1*-deficient mice (Fig. 6d–f). The in vitro data further indicated that FSTL1 promoted monocyte migration (Fig. 6g, h) and upregulated CCR2 expression at 48 h after treatment (Fig. 6i–k). In addition, we hypothesized that FSTL1 increased CCR2 expression by increasing CCR2 stability (Fig. 6l, Supplementary Fig. 9n,). We then adoptively transferred CD45.1^+^ bone marrow cells into the wild-type, FSTL1-pretreated, and FSTL1 + PF4136409-pretreated groups. We observed that FSTL1 pretreatment upregulated CD45.1^+^ infiltrating macrophages and the Ly6C^+^ subset, which was blocked after PF4136409-combined treatment, suggesting that FSTL1 promoted macrophage recruitment in a CCR2-dependent manner (Supplementary Fig. 9j-m). Overall, these data suggest that FSTL1 promotes Ly6C^+^ macrophage recruitment to the fibrotic liver by upregulating CCR2 expression.

### FSTL1 inhibits CCR2 lysosomal degradation and increases CCR2 recycling to the membrane by downregulating *ATP6V1G2* expression in a CD14/TLR/NF-κB-dependent manner

**Fig. 7:**
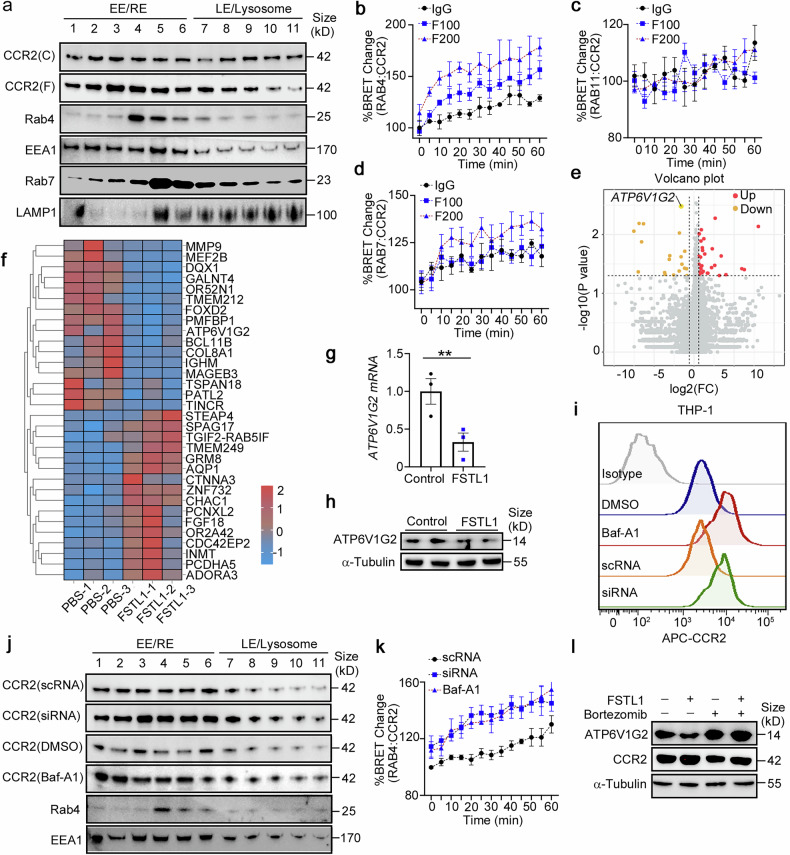
FSTL1 increases CCR2 recycling to the membrane by downregulating *ATP6V1G2* expression in a CD14/TLR/NF-κB-dependent manner. **a** THP-1 cells were subjected to cell fractionation analysis at 48 h after FSTL1 treatment (100 ng/ml). Western blot analysis of CCR2 and endosome markers (Rab4, Rab5, Rab11, EEA1, and Lamp1). **b**–**d** HEK293 cells were transfected with the BRET donor CCR2-Nanoluc along with the BRET acceptor per well (HaloTag-Rab4, HaloTag-Rab11 or HaloTag-Rab7). The cells were treated with or without FSTL1 (100 ng/ml) for 20 h. CCL2 (100 ng/ml) was added and kinetic measurements of donor emission (460 nm) and acceptor emission (618 nm) were collected every 5 min for 60 min. **e**, **f** RNAseq analysis of THP-1 cells treated with or without FSTL1 for 12 h. Gene differential analysis is shown as a volcano plot (**e**) or heatmap (**f**). ATP6V1G2 expression was evaluated via qPCR (**g**, *n* = 3) or western blotting (**h**). **i** FCM analysis of CCR2 expression after 48 h of different treatments. **j** Cell fractionation analysis of CCR2 via western blotting in THP-1cells. **k** NanoBRET assays were used to evaluate the distribution of CCR2 in the recycling or degradation pathway in HEK293 cells treated with or without FSTL1 (100 ng/ml). **l** CCR2 expression was evaluated via western blotting after NF-κB inhibitor (bortezomib) treatment. EE, early endosome; RE, recycling endosome; LE, late endosome. **p < 0.01. Statistical significance was determined by a two-tailed unpaired *t*-test (**g**). Data are presented as the mean ± SEM and were pooled from at least three independent experiments

As internalized CCR2 can be recycled to the membrane or become downregulated through lysosomal degradation,^38^ we next explored whether FSTL1 facilitates the recycling or inhibits the degradation of CCR2 to increase its stability. For this purpose, we characterized the intracellular distribution of CCR2 via cell fractionation.^39,40^ As shown in Fig. 7a, FSTL1 increased the distribution of CCR2 in recycling endosomes (RE, fractions 4–6) but did not obviously affect late endosomes/lysosomes (LEs/Lys, fractions 7–11). We further evaluated the dynamic distribution of CCR2 in the HEK293 cell line via the NanoBRET assay.^41^ As shown in Fig. 7b–d, FSTL1 facilitated CCR2 distribution in Rab4^+^ fast-recycling endosomes (Fig. 7b, c), and enhanced CCR2 distribution in Rab7^+^ LEs/Lys at a relatively high concentration (200 ng/ml, Fig. 7d). Next, we sought to identify FSTL1 target genes via quantitative RNA sequencing. We compared the gene profiles of THP-1 cells treated with or without FSTL1 and detected the downregulation of *ATP6V1G2* (which encodes a G2 subset of V-ATPase) in the FSTL1-treated group (Fig. 7e, f). We confirmed this downregulation after FSTL1 treatment at the mRNA (Fig. 7g) and protein levels (Fig. 7h). As V-ATPase is responsible for the lysosomal function and degradation of CCR2, we hypothesized that the increased fast recycling of CCR2 by FSTL1 is the result of lysosomal acidification and degradation disorders. After treatment with an acidification inhibitor (bafilomycin A1, 0.5 nM) or transfection with *ATP6V1G2 siRNA*, we observed an increase in CCR2 expression on the cell membrane (Fig. 7i) and Rab4^+^-related fast recycling of CCR2 (Fig. 7j, k). It was reported that FSTL1 can bind to CD14 to activate TLR4/NF-κB in monocytes/macrophages and to mediate the inflammatory response.^42^ Here, we found that an NF-κB inhibitor (bortezomib, 10 nM) blocked FSTL1-induced *ATP6V1G2* downregulation in the THP-1 cell line (Fig. 7l). Collectively, these data suggest that FSTL1 decreases CCR2 lysosomal degradation and increases CCR2 recycling to the membrane via transcriptional downregulation of *ATP6V1G2* expression in a CD14/TLR/NF-κB-dependent manner.

### Administration of FSTL1 rescues the therapeutic effect of stem cells in *Fstl1*^+/−^ mice

**Fig. 8:**
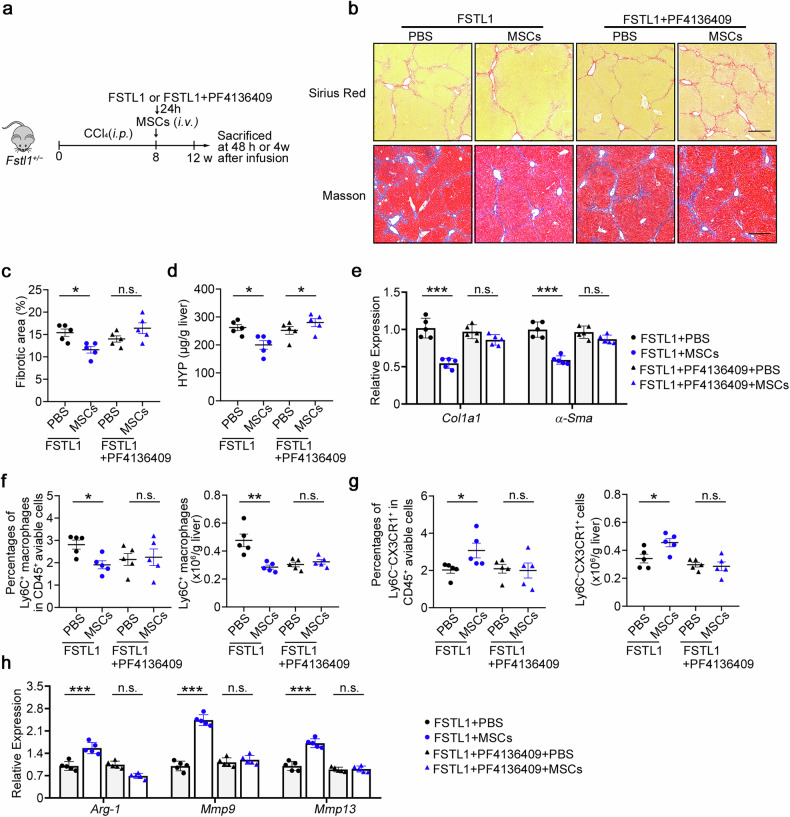
Administration of FSTL1 rescues the defective therapeutic effect of MSCs in *Fstl1*^*+/*−^ mice. **a** Schematic illustration of hepatic fibrosis model establishment and the MSC-based early treatment strategy. **b** The fibrosis degree was evaluated in *Fstl1*^*+/*−^ mice treated with FSTL1 (*n* = 5) or FSTL1 + PF4136409 (*n* = 5) at 4 weeks postinfusion. Liver sections were stained with Sirius red or Masson’s trichrome. Representative images of the staining are shown. Bars, 200 µm. **c** Liver fibrosis score analysis of Sirius red-stained liver sections. The fibrotic area is presented as a percentage. **d** The concentrations of hydroxyproline (HYP) in liver homogenates were determined. **e** Total liver RNA was extracted and the expression of *Col1* and *α-Sma* was determined by qPCR. **f**–**h** Macrophage infiltration was evaluated in *Fstl1*^*+/−*^ mice treated with FSTL1 (*n* = 5) or FSTL1 + PF4136409 (*n* = 5) at 48 h post infusion. **f** The percentages of F4/80^+^CD11b^+^ cells among the total number of viable CD45^+^ cells and the number of CD11b^+^F4/80^+^ cells normalized to the liver weight were determined by flow cytometry. (**g**) The percentages of Ly6C^−^CX3CR1^+^ cells among the total number of viable CD45^+^ cells and the cell counts of the Ly6C^−^CX3CR1^+^ subset were compared. (**h**) F4/80^+^ macrophages were sorted from the livers of *Fstl1*^*+/*−^ mice treated with FSTL1 or FSTL1 + PF4136409 at 24 h post infusion, and the mRNA levels of inflammatory markers (*Arg-1*, *iNOS*) and *Mmp9* were determined by qPCR. *p < 0.05, **p < 0.01, and n.s. not significant. Statistical significance was determined by one-way ANOVA with Tukey multiple comparison test (**c**, **d**, **f**, **g**) or two-way ANOVA with Tukey multiple comparison test (**e**, **h**). Data are presented as the mean ± SEM and were pooled from at least three independent experiments

The above data showed that the administration of FSTL1 to *Fstl1*^*+/−*^ mice increased inflammatory macrophage infiltration and inflammation in the liver. We induced liver fibrosis in *Fstl1*^+/−^ mice and treated them with recombinant FSTL1 protein or FSTL1 + PF4136409 before MSC transplantation (Fig. 8a). Liver fibrosis levels were further evaluated at 4 weeks after MSC transplantation. Sirius red and Masson staining of liver sections (Fig. 8b, c), HYP levels (Fig. 8d) and fibrotic gene expression in liver tissue (Fig. 8e) indicated that MSCs significantly ameliorated liver fibrosis in *Fstl1*^+/−^ mice after FSTL1 addition, whereas FSTL1 + PF4136409 addition in *Fstl1-*deficient mice imparted no further functional benefit after cell infusion. Flow cytometry analysis revealed that FSTL1 addition significantly rescued the immunosuppressive capacity of MSCs in *Fstl1*^+/−^ mice, as indicated by reduced macrophage infiltration (Fig. 8f) and an increased restorative proportion (Fig. 8g), and signature gene expression indicated a shift in the overall macrophage subtype (Fig. 8h), the function of which was blocked by PF4136409. In conclusion, the results indicate that the addition of FSTL1 rescues the therapeutic effect of stem cells in *Fstl1*^+/−^ mice in a CCL2/CCR2 dependent manner.

## Discussion

The therapeutic efficacy of stem cells administered to patients with liver fibrosis is not as robust as that demonstrated in preclinical studies. However, therapeutic mechanisms and reliable potential biomarkers for predicting the immunosuppressive efficacy of stem cell therapy in the clinical setting are still lacking. The results reported in this study prompted us to propose a model of stem cell treatment efficacy under different inflammatory conditions in fibrosis. When stem cells are infused under relatively high inflammatory conditions (high FSTL1 levels), stem cell-mediated Ly6C^+^ profibrotic macrophage recruitment is facilitated by FSTL1, which empowers stem cells to gain an immunosuppressive function, leading to Ly6C^−^CX3CR1^+^ subset accumulation and good antifibrotic effects. However, when infused under mild inflammatory conditions (low FSTL1 levels), however, stem cells fail to acquire immunosuppressive capacity in an FSTL1^low^ microenvironment with insufficient rapid recruitment of Ly6C^+^ macrophages, resulting in insufficient Ly6C^−^CX3CR1^+^ accumulation and poor treatment effects. Mechanistically, FSTL1 facilitates fast recycling of CCR2 via inhibition of *ATP6V1G2* expression and lysosomal degradation of CCR2 through the CD14/TLR4/NF-κB axis. The findings reported in this study provide new insights into the regulatory function of the fibrotic liver microenvironment in stem cell therapy and demonstrate the dual role of FSTL1 as a fibrotic microenvironmental modulator and outcome-predicting functional surrogate of stem cell therapy in liver fibrosis.

Early macrophage recruitment after stem cell transplantation explains the effectiveness of MSCs in chronic inflammatory diseases such as fibrosis. The production of ample amounts of chemokines by MSCs coordinates their immunosuppressive effects on immune cells.^16^ CCL2 is expressed in in vitro-expanded MSCs and further induced by inflammatory cytokines.^43^ An acute immune response characterized by the temporal induction of CCR2^+^ and CX3CR1^+^ macrophages underlies the benefit of cardiac stem cell therapy.^10^ Here, we provide direct in vivo evidence that MSC-induced rapid inflammatory macrophage recruitment is essential for the therapeutic effect of stem cells in liver fibrosis. Aerobic glycolysis sustains the immunosuppression of MSCs.^44^ We found that MSCs cocultured with BMDMs significantly upregulated glycolysis-related, hypoxia-related, and immunosuppressive genes in MSCs. In *Fstl1*-defective mice, recruitment was delayed at 48 h after cell infusion, resulting in enhanced inflammation in MSC-treated mice.

The Ly6C^−^CX3CR1^+^ subpopulation is comprised of potential effector macrophages that accumulate after MSC transplantation. Previously, the effective macrophage subset involved in MSC-mediated antifibrotic therapy has been inconclusive. Anti-inflammatory M2 macrophages and the restorative Ly6C^−^ subset were both used to evaluate the immunoregulatory function of MSCs in liver fibrosis,^6,13^ whereas the CX3CR1 subset is a newly discovered subset according to single-cell RNA sequencing.^10–12^ Although they might inhibit acute liver inflammation, M2 macrophages produce TGF-β during the fibrogenic stage.^45^ In mice, restorative macrophages (Ly6C^−^) highly expressing MMPs participate in fibrosis resolution.^21^ In addition, the CX3CL1–CX3CR1 interaction inhibits inflammatory properties in macrophages, resulting in decreased liver inflammation and fibrosis.^46^ Using single-cell RNA sequencing, we revealed that the potential effector macrophage subset Ly6C^−^CX3CR1^+^ could have both antifibrotic and immunosuppressive functions. In addition, we found that the Ly6C^+^CX3CR1^−^ subset (subset 6) ratio was decreased, whereas the Ly6C^+^CX3CR1^+^ subset (subset 4), which was positive for Cebpβ, was increased in the MSC-treated group. Considering that Ly6C^+^ macrophages are recruited after MSC transplantation, we speculated that Ly6C^+^CX3CR1^+^ macrophages represent an intermediate state between Ly6C^+^ and Ly6C^−^CX3CR1^+^ during MSC-mediated immunosuppression. We propose that the accumulation of the Ly6C^−^CX3CR1^+^ subpopulation is partially due to the increase in Ly6C^+^ macrophage recruitment and the empowerment of stem cell activity after macrophage recruitment.

We propose that FSTL1 is a fibrosis-specific predictor of stem cell-based treatment efficacy in patients with liver fibrosis. We found that the serum level of FSTL1 was greater in patients with liver cirrhosis than in healthy controls, but there was greater variability among patients. Patients with high FSTL1 levels have severe inflammation and improved therapeutic efficacy. Although inflammatory cytokines have long been thought to be important for stem cells,^6,10,47,48^ few studies have reported how disease-specific inflammatory conditions affect treatment effects.^49–52^ Here, we revealed that FSTL1 plays dual roles in the pathology and stem cell treatment of liver fibrosis. Various studies have reported the role of FSTL1 in inflammation and fibrosis.^24,25,27,53^ Consistent with previous findings, we observed a significant reduction in the number of profibrotic Ly6C^+^ macrophages in *Fstl1*^*+/−*^ mice. We also found that FSTL1 is essential for Ly6C^+^ macrophage recruitment and directly influences the MSC-mediated acute immune response. Bone marrow-derived MSCs are isolated from bone marrow MNCs, another adult stem cell type extensively used in human clinical trials, which are composed of multiple stem/progenitor cells, including MSCs. MNC therapy could improve liver function in patients with liver cirrhosis.^54–56^ Our data suggest that FSTL1 also plays an indispensable role in mediating the immunosuppressive capacity and antifibrotic effects of MNCs during therapy.

Cirrhosis-associated inflammation accompanied by different monocyte/macrophage phenotypes and functions likely results in the observed heterogeneity of the therapeutic effect. Monocytes with reduced toll-like receptor 4 (TLR4)^57^ and CCR2^58^ expression are functionally linked to reduced production of TNF-α and IL-6 in response to lipopolysaccharides (LPS) in patients with liver cirrhosis.^22^ FSTL1 has been reported to upregulate the expression of inflammatory factors in monocytes/macrophages via TLR4 signalling.^59^ Our data suggested that FSTL1 upregulated CCR2 expression to promote monocyte recruitment. On the basis of these clinical data, we speculate that high expression of FSTL1 is beneficial for maintaining the function of inflammatory monocytes/macrophages with high TNF-α and IL-6 levels and greater migration ability in responders, which enables stem cells to gain their immunoregulatory function. In clinical treatment, the infusion of stem cells into cirrhotic patients with high inflammation potential may achieve improved efficacy, and the serum FSTL1 concentration can be used as an indicator of both the inflammatory state of patients and the response after treatment. Although FSTL1 is a pivotal molecule that affects liver inflammation and fibrosis, it is not an indicator of disease severity. Patients with end-stage liver disease have immune disorders associated with cirrhosis, that is, systemic inflammation or immune paralysis. The inflammatory pathological features of these two immune states are also completely different.

Although the data suggest that FSTL1 participates in MSC-mediated chemokine–inducible immunosuppression resulting in Ly6C^−^CX3CR1^+^ macrophage subset accumulation, additional evidence for a detailed molecular basis is needed. Recently, MSC apoptosis and phagocytosis by tissue-resident macrophages have been suggested to explain the systemic immunomodulatory effects observed following MSC infusion.^60,61^ However, this is not sufficient to explain the phenotype we observed in *Fstl1-*deficient mice. Further studies are needed to clarify whether MSCs can repair injured tissue by modulating immune responses through the remote secretion of paracrine factors after intravenous injection.^6^ The role of Ly6C^+^/Ly6C^−^ monocytes in the treatment of stem cells has not been fully explored, but we believe that the circulating monocyte state is pivotal for the effect of stem cells in the early stage of infusion. The inflammatory status of monocytes and serum cytokines directly affects the immunoregulatory ability of stem cells, and these stem cell-educated monocytes can engage in chemotaxis to the liver and modulate pathological conditions.

In summary, our findings reveal that FSTL1, which promotes early macrophage recruitment to facilitate subsequent effective macrophage accumulation during stem cell therapy, could potentially serve as a predictive biomarker of the response to stem cell therapy in patients with liver cirrhosis.

## Materials and methods

### Ethics statements

Stem cell transplantation was approved by the Institutional Ethics Committee of Xijing Hospital, The Fourth Military Medical University. Written informed consent was obtained from the participants. The animal study protocol was approved by the Animal Welfare and Ethics Committee of the Fourth Military Medical University and was performed according to the 'Guidelines for the Care and Use of Laboratory Animals'.

### Subjects

Patients with decompensated cirrhosis who received stem cell transplantation were included in the analysis (NCT01728688). Decompensated cirrhosis was diagnosed on the basis of ultrasound or computerized tomography, upper digestive tract endoscopy, and patient history. Chronic hepatitis B was diagnosed on the basis of patient history, serologic tests, and biochemical tests. All patients received standard medical treatment, which consisted of antiviral therapy and the management of complications such as ascites, variceal bleeding, hepatorenal syndromes, and hepatoencephalopathy, according to updated guidelines.^62–64^ In the transplantation group, eligible patients were randomly assigned to receive stem cell treatment. During infusion, the collected cells were transplanted into the recipient's liver via the hepatic artery. Follow-up examinations were routinely performed after treatment. These patients were divided into responsive (*n* = 27) and nonresponsive groups (*n* = 31) according to improvements in the MELD score (MELD change ≤ −10%) at 6 months after treatment.

### Animals

*Fstl1*^*+/−*^ mice were generated as previously described,^65^ and the mice were backcrossed onto the C57BL/6J background for at least 12 generations before use. Six- to eight-week-old male C57BL/6J mice were purchased from Beijing Vitong Lihua Laboratory Animal Technology Co., Ltd. (Beijing, China). All animals were housed and cared for in a pathogen-free airflow cabinet and allowed free access to food and water.

### Liver fibrosis models

Liver fibrosis was induced by the intraperitoneal (i.p.) injection of 7 ml/kg CCl_4_ (20% solution in olive oil) twice per week. Mice were treated with mouse bone marrow-derived MSCs (1 × 10^6^) for early efficacy evaluation at the indicated times. At the designated time points, the mice were euthanized with phenobarbital sodium by i.p. injection, and the livers were harvested for further analyses. Mice with liver fibrosis were treated with IgG or 22B6 (50 µg/each, i.v.), CsA (Selleck, 15 mg/kg, i.p.) or the CCR2 inhibitor PF4136409 (Selleck, final dose of 20 µg each, i.p.) before MSC treatment. *Fstl1*^*+/−*^ mice with liver fibrosis were treated with FSTL1 (2 µg each, i.v.) or FSTL1 (R&D systems, 2 µg each, i.v.) + PF4136409 (final dose of 20 µg each, i.p.) before MSC treatment.

### Flow cytometry

Hepatic mononuclear cells were isolated from the liver with 30% Percoll (GE Healthcare) according to the established method without collagenase digestion.^66^ Single-cell suspensions were first incubated with anti-mouse FcR blocking reagent (BioLegend) and then labelled with the mixed fluorochrome-conjugated antibodies (BioLegend) PerCP/Cyanine5.5 anti-mouse CD45.2, PE/Cyanine 7 anti-mouse F4/80, FITC anti-mouse/human CD11b, APC/Cyanine 7 anti-mouse CX3CR1 and PE anti-mouse Ly6C, APC-conjugated anti-mouse CCR2, APC-conjugated anti-mouse CD45.1, PE-conjugated anti-mouse F4/80, PerCP/Cyanine5.5-conjugated anti-mouse Ly6C, Brilliant Violet 510-conjugated anti-mouse Ly-6G and the Zombie Aqua Fixable Viability Kit (BioLegend). Flow cytometry was conducted with a FACS Canto II flow cytometry system (BD Biosciences). All the data were analysed with FlowJo software (FlowJo LLC, version 10.0.7). Liver macrophages or the Ly6C^–^CX3CR1^+^ subset were sorted from viable CD45^+^F4/80^+^CD11b^+^Ly6G^–^hepatic mononuclear cells with a FACSAria III flow cytometry system (BD Biosciences) for further analysis. Circulating monocyte analysis was performed by staining whole blood after the erythrocytes were lysed.

### Single-cell RNA sequencing (scRNA-seq)

CD45^+^ hepatic mononuclear cells were sorted and the cell suspension was loaded into Chromium microfluidic chips with 3’ (v2 or v3, depending on the project) chemistry and barcoded with a 10× Chromium Controller (10X Genomics). RNA from the barcoded cells was subsequently reverse-transcribed, and sequencing libraries were constructed with reagents from a Chromium Single Cell 3’ v2 reagent kit (10X Genomics) according to the manufacturer’s instructions. Sequencing was performed with an Illumina platform (Nova6000, Novegene, Tianjin, China) according to the manufacturer’s instructions (Illumina)

### Single-cell transcriptome analysis

We use fastp to perform basic statistics on the quality of the raw reads. The raw read sequences produced by the Illumina pipeline in FASTQ format were subsequently preprocessed via Trimmomatic software to obtain clean reads. For the generation and analysis of single-cell transcriptomes, the raw reads were demultiplexed and mapped to the reference genome via the 10X Genomics Cell Ranger pipeline (https://support.10xgenomics.com/single-cell-geneexpression/software/pipelines/latest/what-is-cell-ranger) via default parameters. All downstream single-cell analyses were performed via Cell Ranger and Seurat (Macosko et al., 2015; Satija et al., 2015) unless otherwise mentioned. In brief, for each gene and each cell barcode (filtered by CellRanger), unique molecule identifiers were counted to construct digital expression matrices. Secondary filtration by Seurat was performed as follows: a gene expressed in more than 3 cells was considered expressed, and each cell was required to have at least 200 expressed genes. Some of the foreign cells were filtered out. The Seurat package was used to normalize the data and perform dimensionality reduction, clustering and differential expression. We used the Seurat alignment method for canonical correlation analysis (CCA) [Nat. Biotechnol. 36, 411–420 (2018).] for the integrated analysis of datasets. For clustering, highly variable genes were selected and the principal components based on those genes were used to construct a graph, which was segmented with a resolution of 0.6.

### Cell culture

Mouse bone marrow-derived MSCs was generated from bone marrow from the tibias and femurs of 6- to 10-week-old mice as previously reported.^67^ The cells were cultured in DMEM supplemented with 10% heat-inactivated FBS, 2 mM glutamine, 100 U/ml penicillin, and 100 mg/ml streptomycin (Invitrogen). The cells were used before the 5th passage. Mouse MSCs were used for all MSC therapy mouse models. The characteristics of the MSCs were determined via flow cytometry. The antibodies (anti-CD29, anti-CD44, anti-CD140a, and anti-sca-1) conjugated to PE were purchased from BioLegend (Supplementary Fig. 10a). Osteoblast, adipocyte, and chondrogenic differentiation media were purchased from Cyagen Biosciences (HUXUC-90021, HUXUC-90031, and HUXUC-9004) and used according to the manufacturer’s instructions (Supplementary Fig. 10b). Intracellular lipid or calcium deposits were stained with Oil Red O or Alizarin Red S. The presence of proteoglycans, which indicate chondrogenic differentiation, was verified by toluidine blue staining after 21 d of pellet induction in a 15 mL tube. THP-1, a human leukaemia monocytic cell line, was purchased from ATCC and maintained in RPMI 1640 GlutaMAX medium supplemented with 10% (v/v) heat-inactivated foetal bovine serum (FBS) and a 1% (v/v) mixture of penicillin and streptomycin (all reagents from Thermo Fisher Scientific) in a humidified incubator containing 5% CO_2_ at 37 °C. BMDMs were also generated from the bone marrow of the tibias and femurs of 6- to 10-week-old mice. We obtained nonadherent cells after 12 h of adherent culture and suspended them in BMDM culture medium (DMEM supplemented with 5% fetal bovine serum, and 50 ng/mL M-CSF) at a concentration of 10^7^ cells/mL. Then, the BMDMs were cocultured with or without FSTL1 (100 ng/ml) for 48 h. Chemical inhibitors, Cycloheximide (CHX, 10 μg/ml, Selleck), Baf-A1 (0.5 nM, Selleck) and bortezomib (10 nM, Selleck), were used for inhibiting protein synthesis, blocking lysosome degradation or NF-κB signal transduction.

Mouse MNCs were generated from bone marrow from the tibias and femurs of 14- to 16-week-old mice with established liver fibrosis as previously reported.^10^ Whole bone marrow was first isolated by flushing the dissected femurs and tibiae of mice with liver fibrosis. The suspension was then centrifuged at 400 × *g* for 10 min at 4 °C, resuspended in 3 mL of sterile saline, and layered on top of 4 mL of Ficoll Paque Plus (GE Healthcare). The cells were then centrifuged at 2500 × *g* for 30 min at 4 °C in a swinging bucket rotor centrifuge without brakes. MNCs were isolated by removing of the resulting thin mononuclear cell layer (second layer from the top). Total MNCs were counted with a haemocytometer, washed twice with sterile saline, and resuspended in sterile saline at a final concentration of 1.0 × 10^6^ cells/ml.

### Adoptive transfer of CD45.1^+^ bone marrow cells

We injected CCl_4_-induced CD45.2^+^ recipient mice (including WT and *Fstl1*^*+/*–^, IgG, FSTL1 and FSTL1 + PF4136309) with 5 ×10^6^ bone marrow cells. Then, we analyzed the subsequent distribution of CD45.2^–^CD45.1^+^ donor-derived tissue-resident macrophages (F4/80^+^CD11b^+^) 48 h after bone marrow cell transfer.

### RNA sequencing (RNA-seq) analysis

Total RNA was isolated via TRIzol (Invitrogen) according to the manufacturer’s instructions. RNA sequencing libraries were generated with insert sizes ranging from 370 to 420 bp and sequenced via the Illumina HiSeq 2500 platform (Gene Denovo-Guangzhou, China). The image data measured by the high-throughput sequencer were converted into sequence data (reads) by CASAVA base recognition. Raw data (raw reads) of fastq format were first processed through in-house Perl scripts. In this step, clean data (clean reads) were obtained by removing reads containing adaptors, reads containing N bases and low-quality reads from the raw data. Moreover, the Q20, Q30 and GC contents the clean data were calculated. All the downstream analyses were based on the clean data. Each sample produced an average of 6.0 G of data. The clean reads were mapped to the reference genome with HISAT2 (v2.0.5) software. Data processing and analysis were performed with the R programming language. For quantification of gene expression levels, featureCounts (v1.5.0-p3) was used to count the number of reads mapped to each gene. The expected number of fragments per kilobase of transcript sequence per million base pairs sequenced (FPKM) value of each gene was subsequently calculated on the basis of the length of the gene and the number of reads mapped to that gene. Differential expression analysis of two conditions/groups (two biological replicates per condition) was performed with the DESeq2 R package (1.20.0). DESeq2 provides statistical routines for determining differential expression in digital gene expression data with a model based on the negative binomial distribution. The resulting *P*-values were adjusted with the Benjamini and Hochberg’s approach for controlling the false discovery rate. padj < =0.05 and |log2(foldchange)| >= 1 were set as the thresholds for significantly differential expression. Differentially expressed genes (DEGs) were included for further functional analysis based on Gene Ontology (GO) and Kyoto Encyclopedia of Genes and Genomes (KEGG) databases. The details of all the identified genes are listed in Supplementary Table 2.

### Blood biochemistry measurements

Mouse serum was obtained at each time point, and the levels of alanine aminotransferase (ALT), aspartate aminotransferase (AST) and albumin (ALB) were determined with an automatic biochemistry analyser (Hitachi 7600-120, Hitachi, Japan).

### Hydroxyproline assessment

Hydroxyproline (HYP) levels were assessed with a kit (Jiancheng Bioengineering Institute, Nanjing) following the manufacturer’s protocol. Equal amounts (in weight) of liver tissue samples were also analysed with the kit.

### Cytokine measurements

The human serum concentrations of cytokines (FSTL1, TNF-α, IL-1β and IL-6) were determined using a commercially available Human Magnetic Luminex Assay (R&D Systems) in accordance with the kit-specific protocols provided by the manufacturer. Plates were read on a Bio-Plex 200 system (Bio-Rad Laboratories, Hercules, CA, USA) and analyzed using Bio-Plex Manager software (Bio-Rad Laboratories) with a five-parameter model used to calculate final concentrations and values (expressed in pg/ml). Reference samples were run on each plate to evaluated assay consistency, and all samples were run in a blinded manner. The serum concentrations of cytokines in CCl_4_-induced liver fibrosis model were determined using ELISA kits (FSTL1, Raybiotech; TNF-α, IL-1β and IL-6, Neobioscience).

### Cell fractionation

The cell homogenates were fractionated on Percoll gradients essentially as previously described. Briefly, the cells were rinsed twice with PBS supplemented with 2 mM EDTA and 5 mM EGTA, resuspended in ice-cold homogenization buffer (HB), which consisted of 10 mM HEPES (pH = 7.5), 0.25 M sucrose, 1 mM EDTA, 0.2 mM phenylmethylsulfonyl fluoride (PMSF), and 1 M leupeptin, and homogenized with 22 strokes of a Dounce homogenizer. The homogenate was diluted with an equal volume of fresh HB and centrifuged at 400 × *g* for 10 min at 4 °C to precipitate unbroken cells and nuclei. Postnuclear supernatants were adjusted to a final concentration of 27% Percoll–0.25 M sucrose using a 90% Percoll stock solution and were then layered over a 0.5 ml sucrose cushion consisting of 10x HB. The gradients were centrifuged for 90 min at 25,000 × g in a fixed-angle rotor without braking. A total of 15 fractions were collected manually, starting from the top of the gradient, and 1–11 fractions were collected for analysis via western blotting.

### NanoBRET assays

HEK293 cells were seeded into 6-well plates (8 × 10^5^ cells per well) and transfected with FuGENE HD transfection reagent (Promega) when the cells reached ~70% confluency. The cells were transfected with a BRET donor (Promega, 200 ng of CCR2-Nanoluc per well) along with 2 µg of BRET acceptor per well (Promega, for example, HaloTag-Rab4, HaloTag-Rab11 or HaloTag-Rab7). After 20 h, the transfected HEK293 cells were replated into 96-well plates, the cells were divided into two groups, and HaloTag NanoBRET 618 Ligand or DMSO vehicle was added. The samples were treated with or without FSTL1 (100 ng/ml) for at least 16 h. Live-cell kinetic detection was performed via the NanoBRET Nano-Glo® Kinetic Detection System, and a 1x solution of Nano-Glo Vivazine substrate (a 1:100 dilution of the stock reagent) was prepared in Opti-MEM® I Reduced Serum Medium, with no phenol red + 4% FBS substrate. Vivazine solution was added to each well, and the plate was incubated for 30–60 min at 37 °C and 5% CO_2_ to equilibrate the substrate luminescence. CCL2 (100 ng/ml) was added and kinetic measurements of donor emission (460 nm) and acceptor emission (618 nm) were collected every 3 ~ 5 min via a NanoBRET PPI Assay-compatible luminometer (Promega Biotech, Beijing, China). BRET measurements were taken ~5 min apart for 60 min.

### Statistical analysis

The quantitative data are presented as the mean ± standard error of the mean (SEM). For comparisons between two groups, a two-tailed Student’s t-test was used; when the data were not normally distributed, a nonparametric Mann–Whitney U-test was used. For comparisons across multiple groups, one-way analysis of variance (ANOVA) with Tukey multiple comparison test was used.

Comparisons between the same individual were performed with Wilcoxon’s matched-pairs test. The relationship between two variables was evaluated with the Spearman rank correlation test. *P*-values of < 0.05 were considered statistically significant. The 'pROC' package was used to plot the ROC curves. The optimum cut-off point was determined by the greatest Youden index, and the sensitivity and specificity were calculated on the basis of the cut-off point. All the statistical analyses were performed with GraphPad Prism 8.0 (GraphPad Software, CA), SPSS software version 21.0 (SPSS Inc.) and R (version 3.6.1).

## Supplementary information

Supplemental Material

Supplemental Material

## Data Availability

The high-throughput sequencing data in this study have been deposited into the Genome Sequence Archive (GSA) and the GSA for Humans with accession numbers CRA021547, CRA021609 and HRA009783. The scRNA-seq data of patients with liver cirrhosis and CCl_4_-induced model mice can be downloaded from the NCBI Gene Expression Omnibus database under accession numbers GSE136103 and GSE171904.
