# Supplementary material for: Host FSTL1 defines the impact of stem cell therapy on liver fibrosis by potentiating the early recruitment of inflammatory macrophages
Source: Signal Transduct Target Ther. 2025 Mar 7;10:81. doi: 10.1038/s41392-025-02162-6 (PMC11885662; doi:10.1038/s41392-025-02162-6)
Supplement: Supplementary file 1 — Supplemental Material [file 41392_2025_2162_MOESM1_ESM.docx]

Supplementary Materials for

Host FSTL1 Defines the Impact of Stem Cell Therapy on Liver Fibrosis by Potentiating the Early Recruitment of Inflammatory Macrophages

Xiaohong Zheng^1*^, Siyuan Tian^1*^, Ting Li^1*^, Si Zhang^2^, Xia Zhou^1^, Yansheng Liu^1^, Rui Su^1^, Miao Zhang^1^, Bo Li^1^, Chao Qi^2^, Guanya Guo^1^, Shuoyi Ma^1^, Keshuai Sun^1^, Fangfang Yang^1^, Yinan Hu^1^, Chunmei Yang^1^, Lina Cui^1^, Yulong Shang^1^, Changcun Guo^1^, Boquan Jin^3^, Lei Guan^1^, Jingbo Wang^1,4#^, Wen Ning^2#^, Ying Han^1#^

^*^These authors contributed equally

^#^Correspondence should be addressed to Ying Han ([hanying1@fmmu.edu.cn](mailto:hanying1@fmmu.edu.cn); hanyingxj@126.com), Wen Ning ([ningwen108@nankai.edu.cn](mailto:ningwen108@nankai.edu.cn)) or Jingbo Wang ([jimberw@163.com](mailto:jimberw@163.com))

**This PDF file includes:**

Figures. S1 to S11

Tables S1

**Figure. S1.**


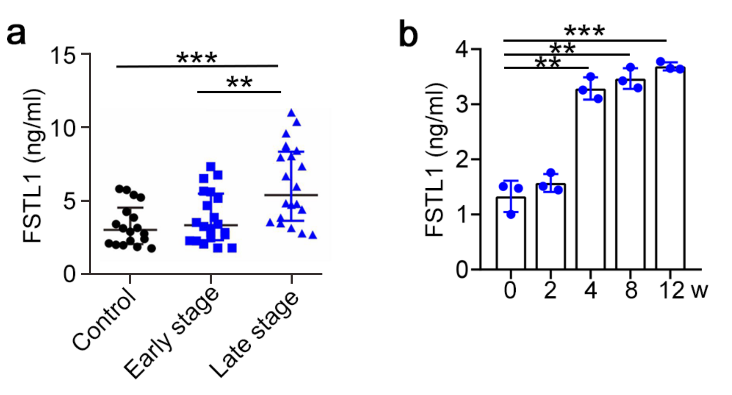


**Figure. S1. Elevated serum FSTL1 in patients with liver cirrhosis and CCl_4_-induced fibrotic mice.** (**a**) ELISA analysis of FSTL1 in serum from healthy control (n =18) and patients with HBV-related liver cirrhosis (Early stage, n =18; Late stage, n=18). (**b**) ELISA analysis of FSTL1 in serum at indicate time after CCl_4_ treatment mice (n=3). **, P<0.01; ***, P<0.001. Statistical significance was determined by Mann–Whitney U-test (**a**) or one-way ANOVA with Tukey multiple comparison test (**b**). Data are presented as means ± SEM and were pooled from at least three independent experiments.

**Figure. S2.**


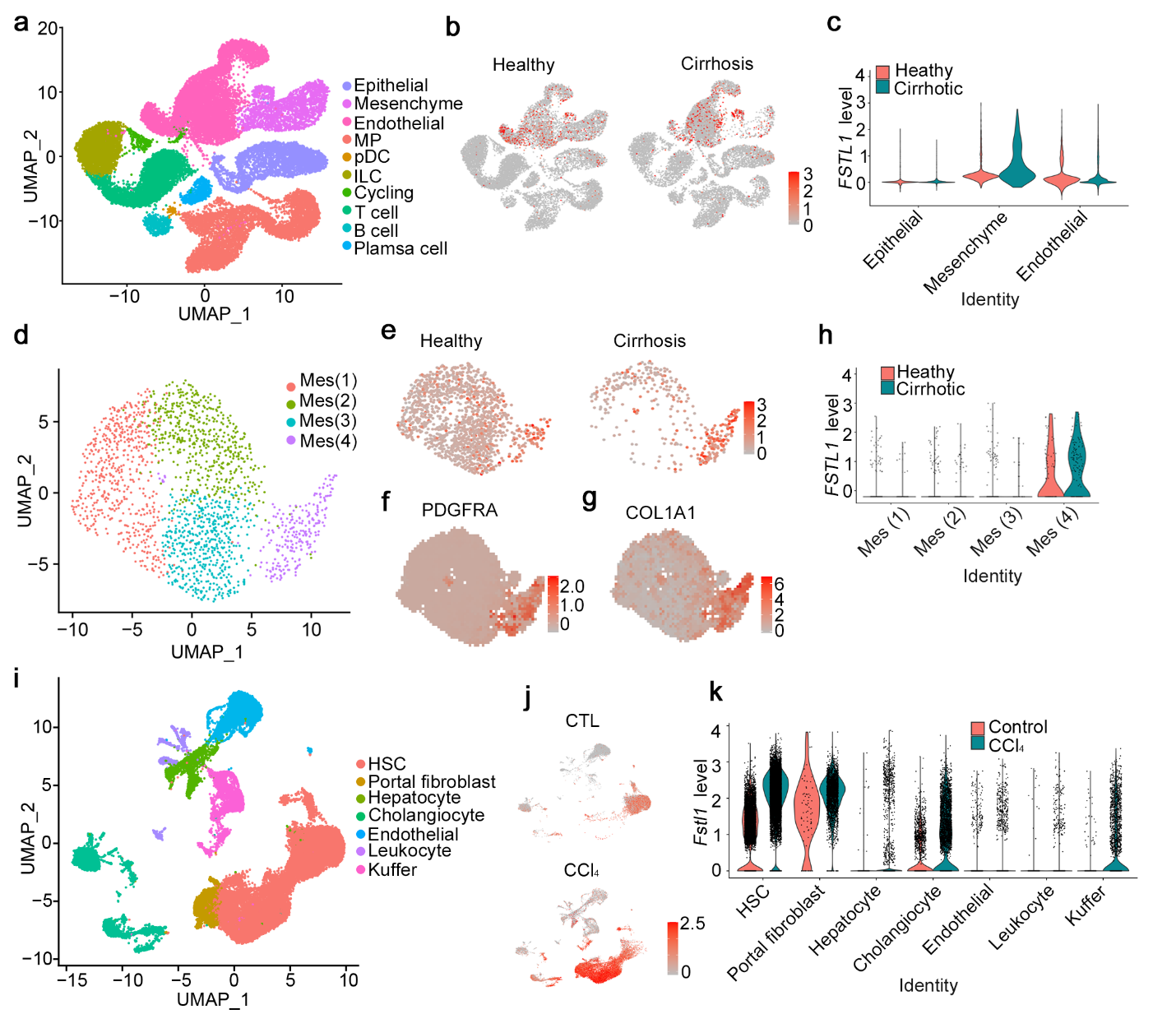


**Figure. S2. Source of FSTL1 in fibrotic liver.** (**a**) Cell lineage inferred from the expression of marker gene signatures in nonparenchymal cell clusters from healthy controls (n=4) and patients with liver cirrhosis (n=3). (**b**) FSTL1 expression in nonparenchymal cell clusters. (**c**) Scaled gene expression of FSTL1 across epithelial, mesenchymal, and endothelial cells. (**d**) Clustering of 2,318 mesenchymal cells (Mes) from 4 healthy and 3 cirrhotic human livers. (**e**) FSTL1 expression in mesenchymal cells. F, G: PDGFA (**f**) and (**g**) COL1A1 expression in mesenchymal cells. (**h**) Scaled gene expression of FSTL1 across mesenchymal cells. (**i**) Cell lineage inferred from the expression of marker gene signatures in nonparenchymal cell clusters from control (10,636) and CCl_4_-induced livers (18,185). (**j**) FSTL1 expression in nonparenchymal cell clusters. (**k**) Scaled gene expression of FSTL1 in nonparenchymal liver cells. MP, mononuclear phagocyte; pDC, plasmacytoid dendritic cell; ILC, innate lymphoid cell; chol, cholangiocytes.

**Figure. S3.**


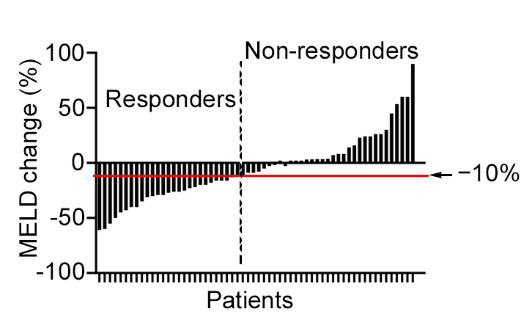


**Figure. S3. Definition of responding patients according to the MELD score improvement.** These patients were divided into the responsive (n=27) and nonresponsive groups (n=31) according to the improvement in MELD score (MELD Changes ≤*−*10%).

**Figure. S4.**


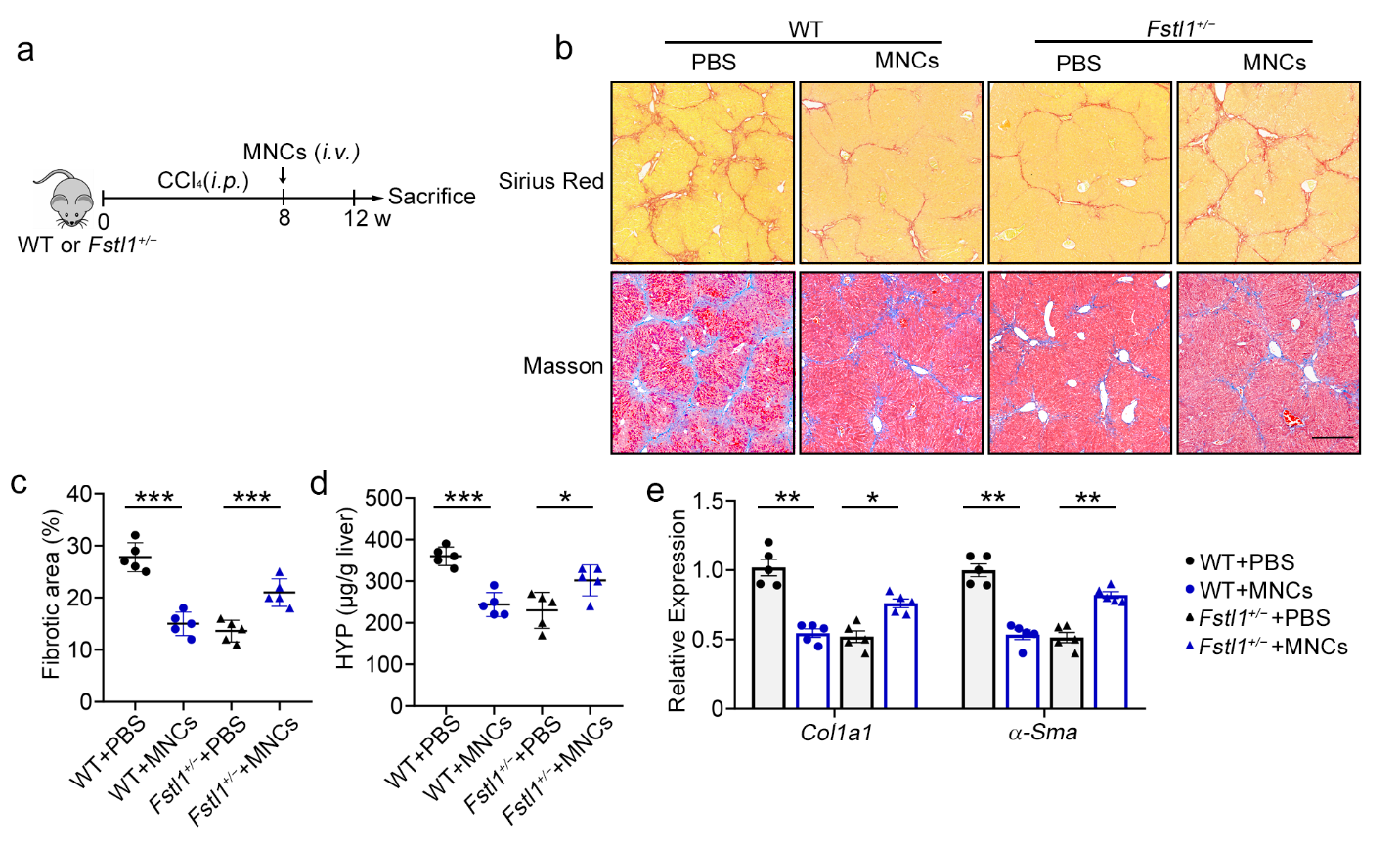
 **Figure. S4. Anti-fibrotic treatment effect of stem cells is abrogated in *Fstl1^+/^*^−^ mice.** (**a**) Schematic illustration of hepatic fibrosis model establishment and the MNC-based treatment strategy. (**b**–**e**) The fibrosis degree was evaluated in *Fstl1^+/^*^−^ (n=5) and WT littermates (n=5) at 4 weeks post cell infusion. (**b**) Liver sections were stained with Sirius red or Masson’s staining. Representative images of the staining are shown. Bars, 200 µm. (**c**) The fibrotic area is presented as a percentage. (**d**) The concentrations of hydroxyproline (HYP) in liver homogenates were determined. (**e**) Total liver RNA was extracted and the expression of *Col1* and *α-Sma* was determined by qPCR. *p<0.05, **p<0.01, ***<0.001. Statistical significance was determined by one-way ANOVA with Tukey multiple comparison test (**c**, **d**) or two-way ANOVA with Tukey multiple comparison test (**e**). Data are presented as the mean ± SEM and were pooled from at least three independent experiments.

**Figure. S5.**


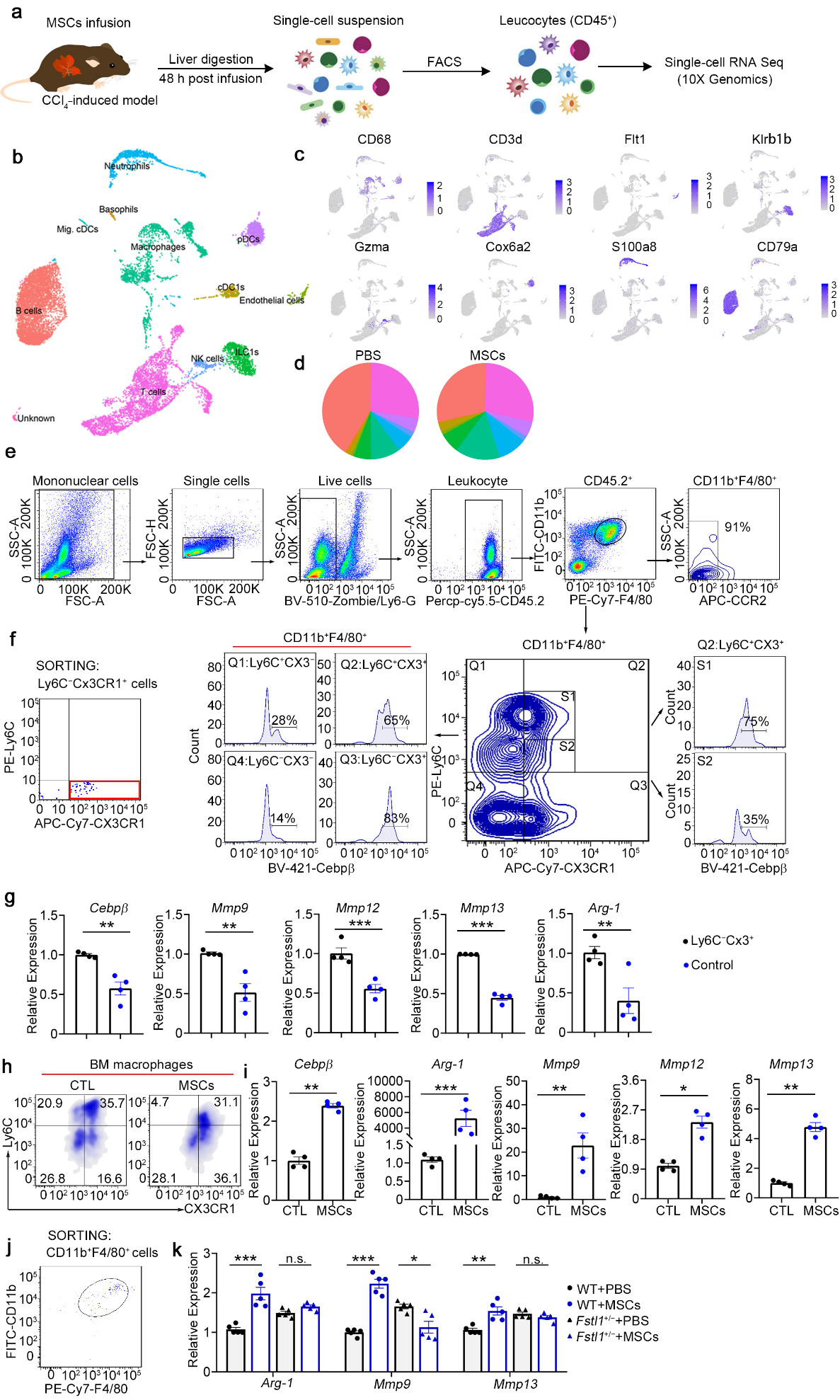


**Figure. S5. Fstl1 facilitates stem cell-meditated Ly6C*^−^*CX3CR1^+^ subsets remodeling. (a)** The strategies for preparing single cells for single-cell RNA sequencing (scRNA-seq). **(b)** UMAP analysis of 15325 liver cells from PBC- and MSCs-treated mice. (**c**) the expression levels of well-known representative enriched marker genes across 11 cell lineages. (**d**) Statistical difference between the two groups in different cell subtypes. (**e**) Gating strategies for Ly6C^−^CX3CR1^+^CCR2^−^ subset. (**f**) Ly6C^−^CX3CR1^+^ sorting. (**g**) Representative genes expression in Ly6C^−^CX3CR1^+^ subset and control (n=4). (**h, i**) Ly6C^−^CX3CR1^+^ subset (**h**) and genes expression (**i**, n=4) was determined in BMDM 48 h post co-cultured with MSCs. (**j**) F4/80^+^CD11b^+^ macrophages sorting. (**k**) Macrophages were isolated from the livers *Fstl1^+/^*^−^ (n=5) and WT littermates (n=5) at 48 h post cell infusion. mRNA levels were determined by qPCR. *p<0.05, **p<0.01, ***<0.001. Statistical significance was determined by a two-tailed unpaired t test (**g, i**) or by two-way ANOVA with Tukey multiple comparison test (**k**). Data are presented as the mean ± SEM and were pooled from at least three independent experiments.

**Figure. S6.**

**Figure. S6. Ly6C^+^ macrophages recruitment post cell infusion facilitates stem cell-mediated Ly6C*^−^*CX3CR1^+^ macrophages remodeling.** (**a**) Peripheral monocytes were evaluated in wildtype at 24 h and 48 h post MSCs infusion by flow cytometry. (**b**) CCL2 level was evaluated by ELISA in supernatant and serum (n=3). *p<0.05, statistical significance was determined by one-way ANOVA with Tukey multiple compari
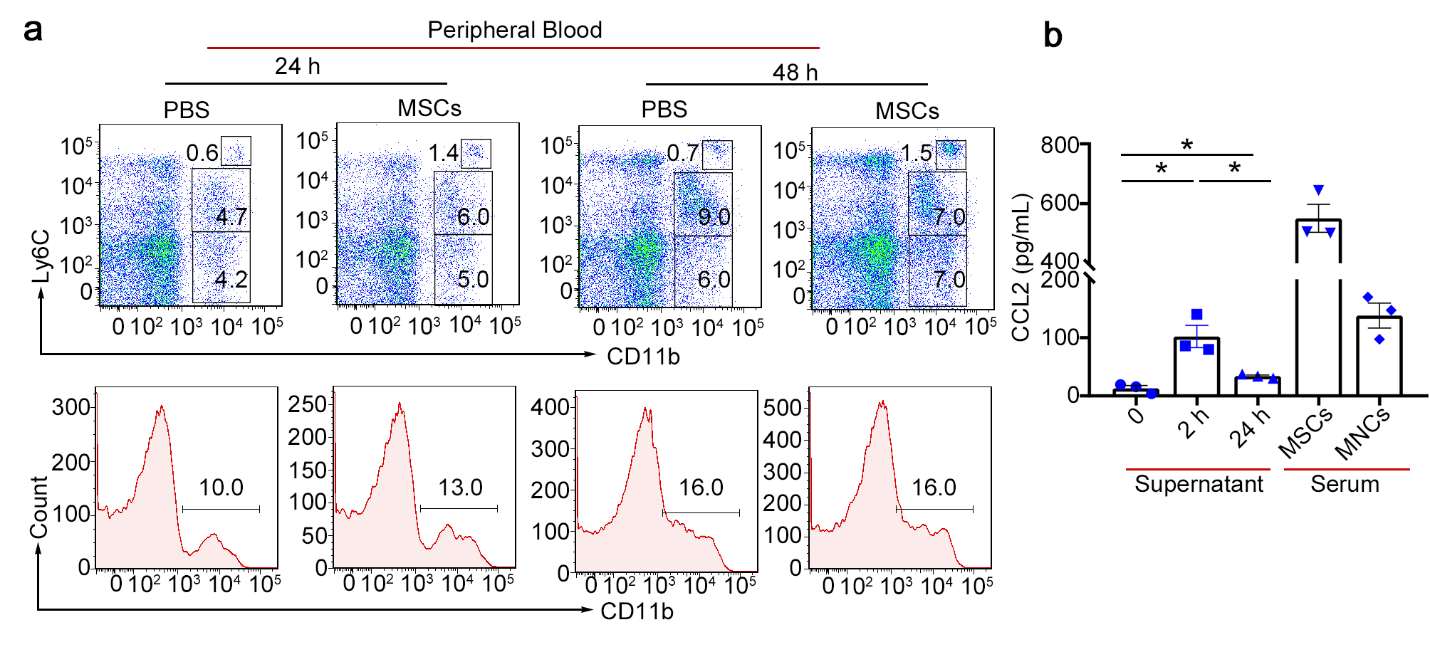
son test. Data are presented as the mean ± SEM and were pooled from at least three independent experiments.

**Figure. S7.**


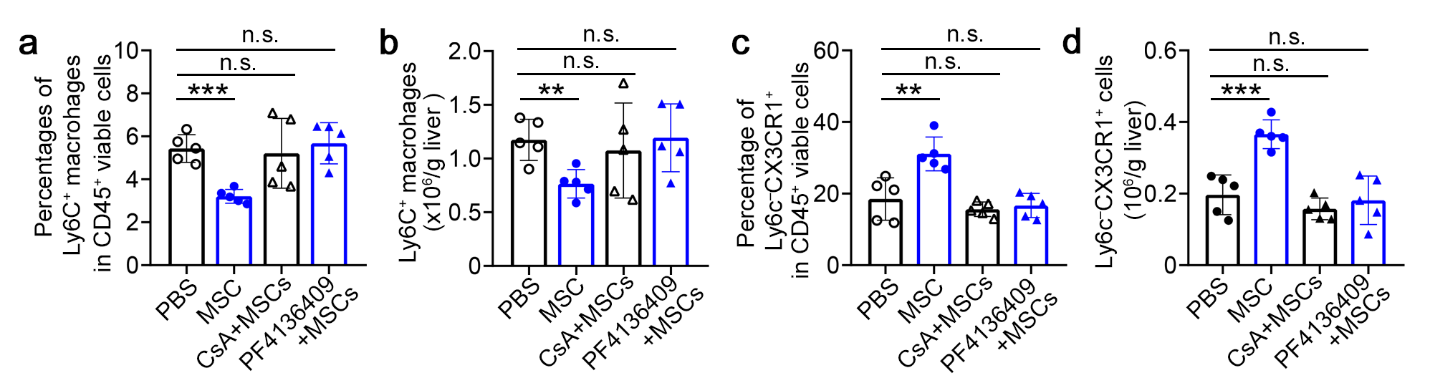


**Figure. S7. The recruitment of Ly6C^+^ macrophages post cell infusion facilitates stem cell-mediated Ly6C*^−^*CX3CR1^+^ macrophage remodeling. (a, b)** Macrophage infiltration was evaluated at 48 h after MSC infusion by flow cytometry. (**a**) The percentages of Ly6C^+^ macrophages in the F4/80 CD11b^+^ population were compared, and (**b**) the cell count was normalized to the liver weight. (**c**) The percentages of the Ly6C^−^CX3CR1^+^ subset among the total viable hepatic CD45^+^ cells and (**d**) the cell counts of the Ly6C^−^CX3CR1^+^ subset normalized by liver weight determined by flow cytometry. **p<0.01, ***<0.001. Statistical significance was determined by one-way ANOVA with Tukey multiple comparison test (**a−d**). Data are presented as the mean ± SEM and were pooled from at least three independent experiments.

**Figure. S8.**
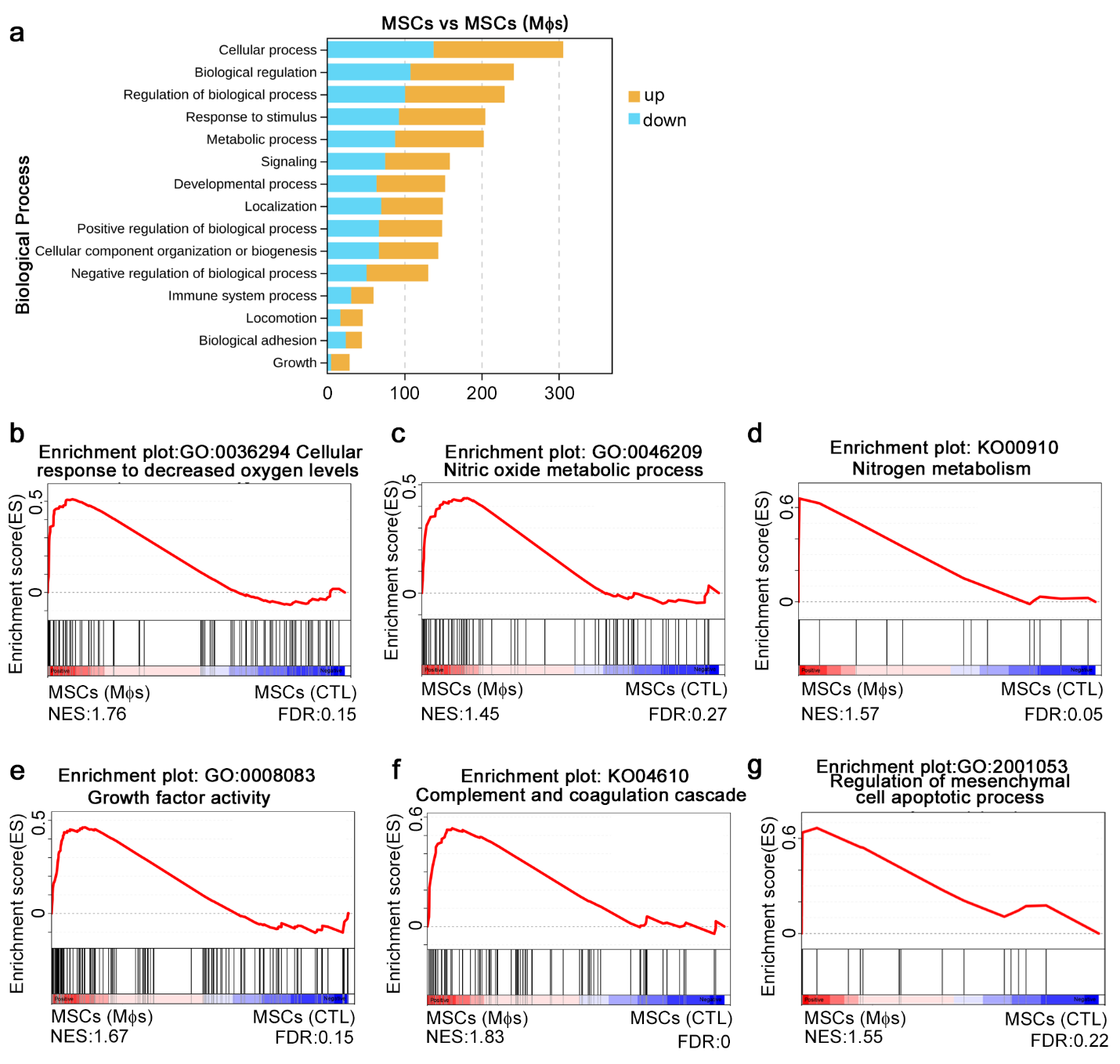


**Figure. S8. Inflammatory macrophages reprogrammed metabolism and improved immunosuppressive capacity of MSCs. (a)** Cluster analysis of differential genes related to biological process in GO were shown. **(b−g)** GSEA plots of the RNA-seq data of MSCs co-cultured with BMDM and control MSCs.

**Figure. S9.**


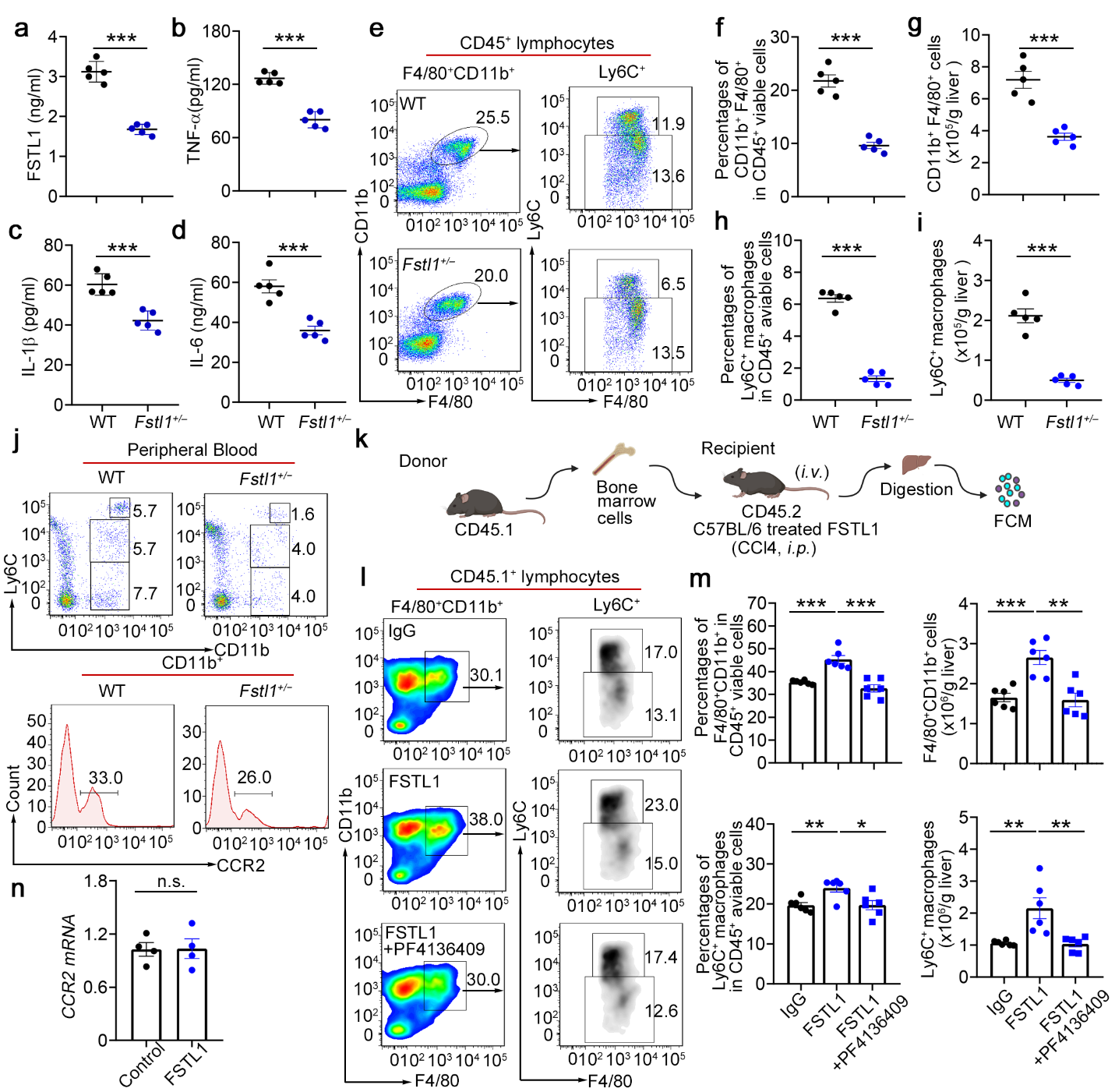


**Figure. S9. FSTL1 potentiates Ly6C^+^ macrophages hepatic recruitment via up-regulating CCR2 expression.** (**a**-**d**) Serum inflammatory cytokines, including (**a**) FSTL1, (**b**) TNF-α, (**c**) IL-1β, (**d**) IL-6 in wild-type (n=5) and *Fstl1^+/−^* mice (n=5). (**e−i**) Macrophage infiltration was evaluated by flow cytometry in C57BL/6 (n=5) 8 weeks post CCl4 induction in *Fstl1^+/^*^−^ and wild-type littermates. (**e, f**) The percentages of F4/80^+^CD11b^+^ cells among the total viable CD45^+^ cells and (**g**) the cell counts normalized by liver weight determined. (**h**)The percentages of Ly6C^+^macrophages cells among the total viable CD45^+^ cells and (**i**) the cell counts normalized by liver weight determined. (**j**) The percentages of CD11b^+^Ly6C^+^ cells among the total viable CD45^+^ cells were determined. (**k**) Schematic illustration of adoptive transfer of CD45.1 bone marrow cells into FSTL1- or FSTL1+PF4136409-treated mice. (**l**, **m**) CD45.1^+^ infiltrated macrophages and CD45.1^+^Ly6C^+^ macrophages were evaluated 48 h post injection (n=6). (**n**) *CCR2* *mRNA* evaluated via qPCR (n=4). *p<0.05, **p<0.01, ***<0.001 and n.s., not significant. Statistical significance was determined by a two-tailed unpaired t test (**a−d, f−i, n**), by one-way ANOVA with Tukey multiple comparison test (**m**). Data are presented as the mean ± SEM and were pooled from at least three independent experiments.

**Figure. S10.**


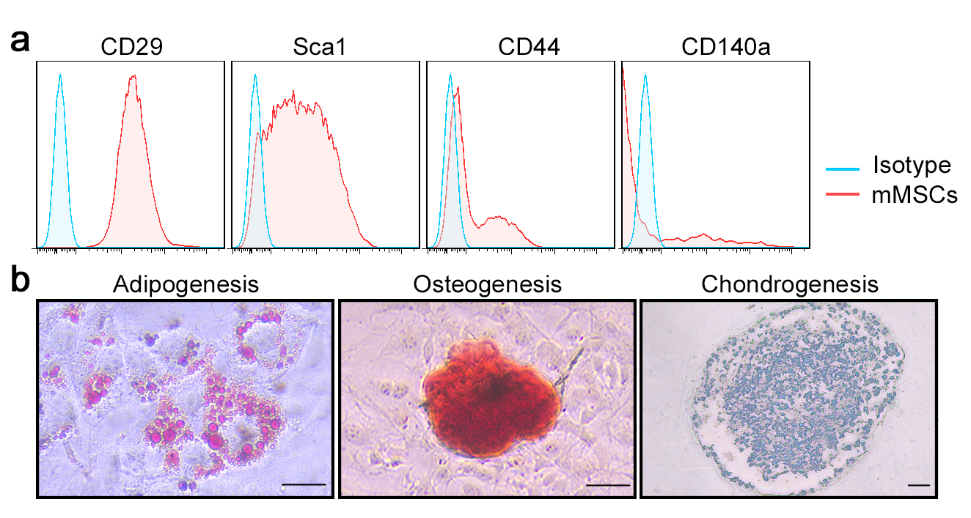


**Figure. S10. Mouse BM-derived MSC identification.** (**a**) Expression of CD molecules on cell surface; (**b**) Assessment the adipogenic , osteogenic and chondrogenic abilities of mouse BM-derived MSCs. Bars, 50 µm.

**Figure. S11.**


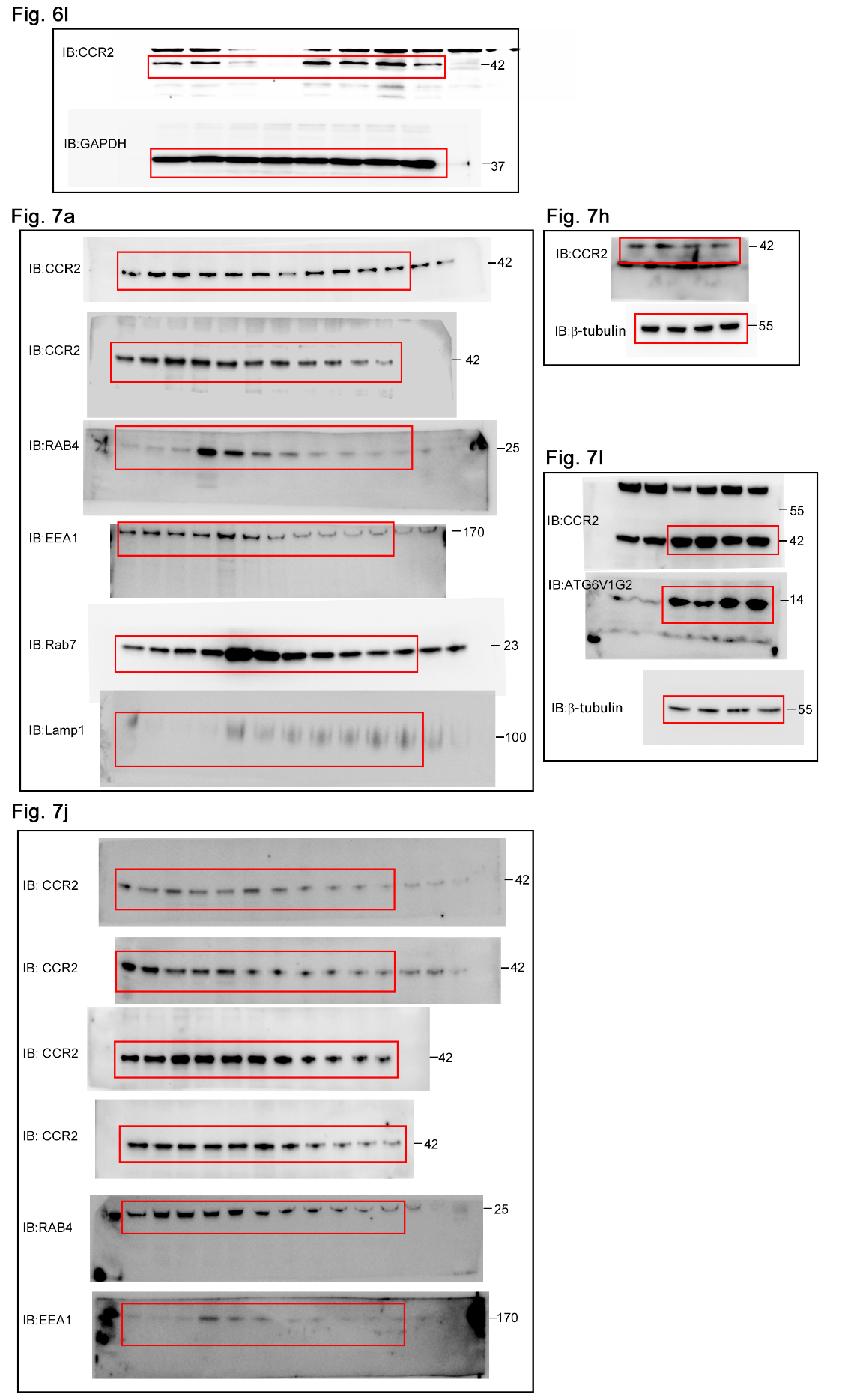


Table S1.

**Baseline characteristics of patients with MSCs-responders and non-responders**

|  | Cirrhosis | Responders | Non-Responder | *P* value |
| --- | --- | --- | --- | --- |
| Number | 58 | 27 | 31 |  |
| Male/Female, n (%) | 40/18 (69.0/31.0) | 17/10 (63.0/37.0) | 23/8 (74.2/25.8) | 0.404 |
| Age, years | 45.2±9.12 | 44.9±7.7 | 45.6±10.2 | 0.503 |
| Ascites, n (%) | 39 (67.2) | 19 (70.4) | 20 (64.5) | 0.781 |
| Albumin, g/L | 28.1 (25.9-31.5) | 27.1 (25.6-31.6) | 29.5 (27.1-31.6) | 0.234 |
| ALT (U/L) | 35.5 (22.0-44.8) | 38.0 (23.2-53.8) | 32.0 (21.5-43.3) | 0.501 |
| AST (U/L) | 50.5 (35.8-58.0) | 52.5 (39.0-73.5) | 40.5 (33.0-54.3) | 0.129 |
| GGT (U/L) | 30.5 (20.0-69.5) | 27.0 (16.0-56.0) | 38.0 (21.8-77.3) | 0.309 |
| Bilirubin, μmol/L | 29.6 (20.4-52.5) | 40.1 (20.7-65.5) | 27.9 (19.8-41.3) | 0.157 |
| Creatinine, μmol/L | 75.0 (70.0-84.0) | 75.0 (66.8-83.0) | 75.0 (72.0-92.0) | 0.227 |
| Prothrombin time (s) | 18.2 (17.4-21.7) | 21.2 (18.7-22.8) | 17.6 (16.6-18.3) | 0.003 |
| MELD score | 11.6 (9.4-14.9) | 14.7 (10.0-16.9) | 10.7 (8.1-12.9) | 0.016 |
| Child-Pugh score | 9.0 (7.0-11.0) | 10.5 (8.0-11.8) | 8.0 (6.0-9.0) | 0.008 |
